# Adhesins and Host Serum Factors Drive Yop Translocation by *Yersinia* into Professional Phagocytes during Animal Infection

**DOI:** 10.1371/journal.ppat.1003415

**Published:** 2013-06-20

**Authors:** Francisco J. Maldonado-Arocho, Carlos Green, Michael L. Fisher, Michelle K. Paczosa, Joan Mecsas

**Affiliations:** 1 Tufts University School of Medicine, Boston, Massachusetts, United States of America; 2 Sackler School of Biomedical Sciences, Boston, Massachusetts, United States of America; Stanford University School of Medicine, United States of America

## Abstract

*Yersinia* delivers Yops into numerous types of cultured cells, but predominantly into professional phagocytes and B cells during animal infection. The basis for this cellular tropism during animal infection is not understood. This work demonstrates that efficient and specific Yop translocation into phagocytes by *Yersinia pseudotuberculosis* (*Yptb*) is a multi-factorial process requiring several adhesins and host complement. When WT *Yptb* or a multiple adhesin mutant strain, *ΔailΔinvΔyadA*, colonized tissues to comparable levels, *ΔailΔinvΔyadA* translocated Yops into significantly fewer cells, demonstrating that these adhesins are critical for translocation into high numbers of cells. However, phagocytes were still selectively targeted for translocation, indicating that other bacterial and/or host factors contribute to this function. Complement depletion showed that complement-restricted infection by *ΔailΔinvΔyadA* but not WT, indicating that adhesins disarm complement in mice either by prevention of opsonophagocytosis or by suppressing production of pro-inflammatory cytokines. Furthermore, in the absence of the three adhesins and complement, the spectrum of cells targeted for translocation was significantly altered, indicating that *Yersinia* adhesins and complement direct Yop translocation into neutrophils during animal infection. In summary, these findings demonstrate that in infected tissues, *Yersinia* uses adhesins both to disarm complement-dependent killing and to efficiently translocate Yops into phagocytes.

## Author Summary

Many bacterial pathogens use a needle-like structure to deliver proteins into host cells to cause disease. *Yersinia* species use one such structure, called a type III secretion system, to deliver a set of 6–7 proteins, called Yops, into host cells. These Yops act to dismantle host defenses and establish infection. Bacterial adhesins and host factors have been suggested to promote proper delivery of Yops into specific mammalian cells. We identify three *Yersinia pseudotuberculosis* adhesins that significantly contribute to bacterial survival and efficient Yop delivery into host cells during animal infection. We also demonstrate that host serum factors in combination with *Yersinia* adhesins contribute to the number of cells that are injected with Yops and to the specific cell types targeted for injection. Our study illustrates that bacterial adhesins and host factors contribute to efficient delivery of effector proteins into targeted host cells during infection.

## Introduction

Translocation of effectors via a type III secretion system (TTSS) is an essential process used by many gram-negative bacterial pathogens to thwart immune defenses during infection [1]. Upon mammalian infection, the three pathogenic *Yersinia spp.*, *Yptb*, *Y. enterocolitica* and *Y. pestis* deliver 5–6 Yop effectors into cells of the innate immune system [2]–[4]. Most Yops target and disrupt functions of macrophages, neutrophils and dendritic cells [5]–[8]. While Yop delivery is crucial for the virulence of *Yersinia spp.* [9], the molecular interactions driving Yop translocation into innate immune cells during tissue infection are not understood.

Bacterial attachment to host cells is necessary for TTSS-mediated delivery of effector proteins, and several studies have suggested a role for adhesins in attachment and TTSS mediated delivery by Enteropathogenic *Escherichia coli*, *Pseudomonas* and *Yersinia spp.* into host cells [5], [10]–[16]. *Yersinia* expresses numerous adhesins, including Ail, Invasin and YadA, all of which can promote Yop translocation into cultured cells [11], [16]. Invasin and YadA are expressed by the two enteric *Yersiniae*, are important for colonization, and contribute to dissemination following oral inoculation [17]–[19]. Ail is expressed by all three pathogenic *Yersinia spp.* and facilitates Yop delivery by *Y. pestis* into human epithelial and monocytic cell lines [16], [20]. Ail and YadA are also implicated in conferring serum resistance [21], [22], and Ail, Invasin and YadA promote invasion into cultured cells [23]–[25]. However, while informative, cell culture and *in vitro* systems do not fully recapitulate the interactions between *Yersinia* and host cells during the course of infection. For example, while deleting Invasin and YadA is sufficient to abrogate Yop translocation in cell culture models [11], a *ΔinvΔyadA* mutant still translocates effectors into freshly isolated splenocytes [3] and is still virulent in murine infection [26]. Thus, while many of the molecular mechanisms of adhesin functions have been well characterized in cell culture, their roles in Yop translocation and serum resistance during animal infection have not been established.

The presence of multiple adhesins suggests at least four scenarios for their role in *Yptb* pathogenesis. First, expression of specific adhesins may be important at distinct stages of infection. It is established that invasin is necessary for survival in the GI tract and in penetrating the Peyer's patches, but is dispensable for establishing systemic infection [27], [28]. Second, expression of certain adhesins may influence the ability of *Yptb* to disseminate to distinct tissues. In fact, YadA expression contributes to colonization of the lungs following intravenous (IV) infection with *Yptb* [29]. Third, functional redundancy may exist between adhesins, such that eliminating one adhesin may have minimal or no effect on virulence and/or translocation. For example, in the absence of both Invasin and YadA, other *Yersinia* factors enable penetration of *Yptb* across the intestinal epithelium [18], [30]. Fourth, some adhesins may have roles unrelated to cell binding. For instance Ail and/or YadA may function to resist killing by serum during tissue infection [21], [22]. Therefore, expression of multiple adhesins may contribute to *Yptb* survival in distinct host niches; some may direct Yop delivery into cells during infection while others may have different and/or additional roles.

Host-encoded factors also play a part in Yop translocation in both cell culture systems and tissue infections. Activation of host signaling pathways, triggered by binding of Invasin or YadA to β1-integrin, promotes efficient Yop translocation into epithelial cell lines [4], [11]. Antibody or complement opsonization of *Yersinia* is sufficient to drive Yop delivery into phagocytes grown in culture [5], [31]. Moreover, serum factors, such as albumin, can activate TTSS-mediated secretion in *Yersinia* [32], indicating that crosstalk between *Yersinia* and the host leads to proper engagement of the TTSS machinery. Finally, Yop delivery is severely reduced during infection of mice depleted of Gr-1^+^ cells, which include neutrophils and inflammatory monocytes [3], suggesting that *Yptb* recognizes and mediates Yop translocation in response to a specific cellular environment.

Complement is an integral part of the innate immune system and is composed of more than 30 proteins present in serum, tissue fluids and on cell surfaces [33]. By mediating bacteriolysis, opsonization and inflammation, complement acts as a rapid and efficient surveillance system against invading pathogens. Many bacterial pathogens have evolved mechanisms for evading the action of complement by sequestering or degrading complement proteins [34]. *In vitro*, *Yersinia* adhesins Ail and YadA mediate complement resistance by binding complement proteins and rendering them ineffective [21], [22], [35]. This binding prevents killing by the membrane attack complex (MAC); however, mice lack the C5 convertase and thus are unable to kill pathogens via MAC [36], [37]. Binding of complement proteins to *Yptb* may also bridge contact of *Yersinia* with host cells, facilitating Yop translocation. Whether host opsonins participate in *Yptb* binding to and/or Yop delivery into host cells during animal infection remains to be determined.

In this work, we examined the contribution of *Yptb* adhesins and host serum factors to the specific interactions between *Yptb* and immune cells leading to Yop translocation. We find the adhesins Ail, Invasin and YadA, function to direct translocation of Yops in infected tissues. We demonstrate that complement plays a role in limiting infection by *ΔailΔinvΔyadA* indicating that these adhesins also thwart complement-dependent killing. Finally, we show that complement, together with *Yptb* adhesins, promotes Yop translocation into professional phagocytes during animal infection. This illustrates that bacterial and host factors act in concert for efficient Yop delivery into targeted host cells during *Yptb* infection.

## Results

### *Yptb ΔailΔinvΔyadA* mutant is defective in translocating Yops into isolated splenocytes

**Figure 1:**
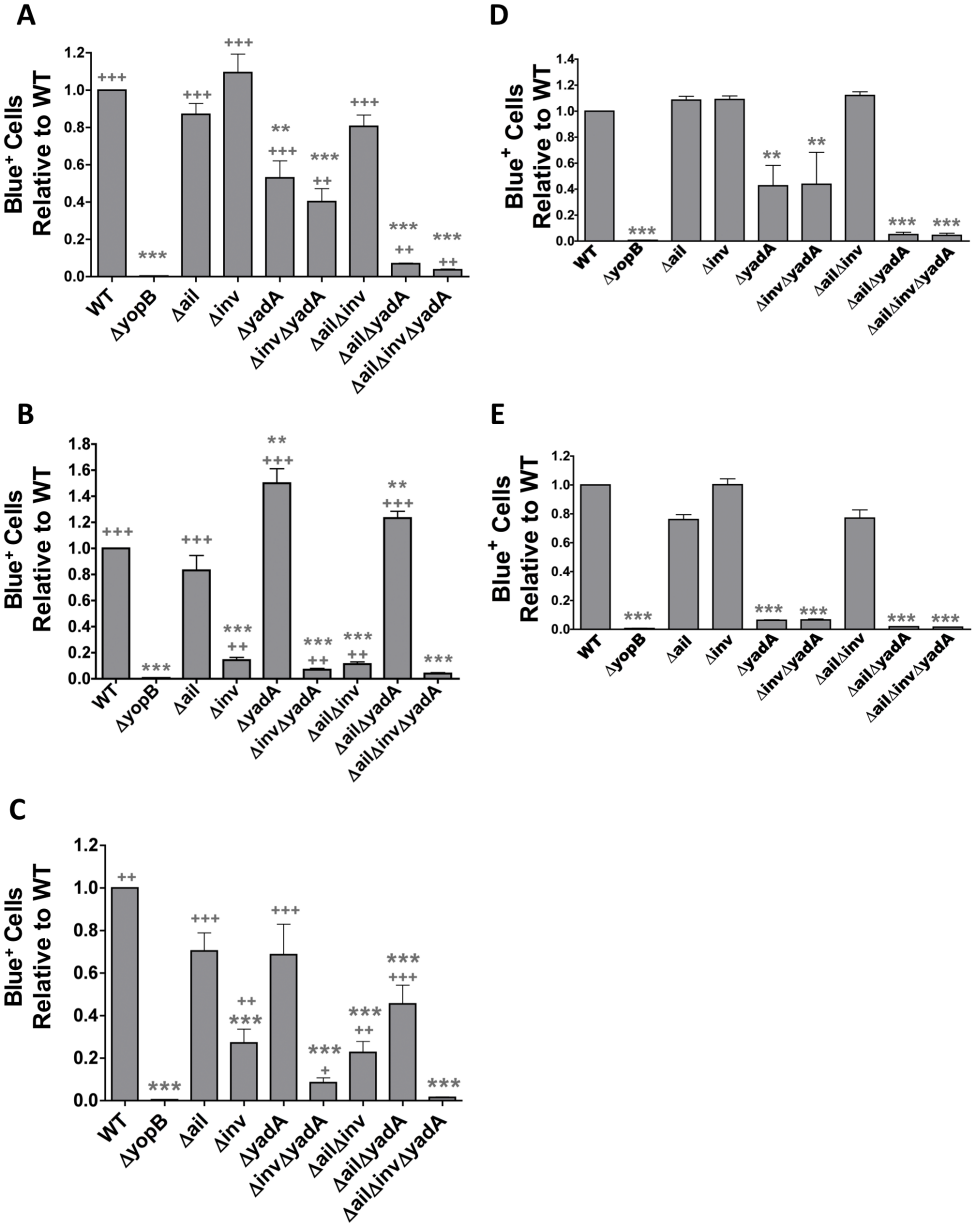
*ΔailΔinvΔyadA* strains are defective for Yop translocation into isolated splenocytes. Splenocytes were infected with the indicated ETEM-expressing strains (**A–C**) at an MOI of 1∶1 for (**A**) 1 h with IP2666 strains, (**B**) 45 min with IP32953 strains or (**C**) 45 min with YPIII strains, or (**D–E**) with the indicated IP2666 strains for (**D**) 4 h at an MOI of 1∶1 or (**E**) 1 h at an MOI of 20∶1. (**A–E**) CCF4 conversion from green to blue was measured by flow cytometry and the relative percentage of Blue^+^ cells was determined by setting WT to 1 and normalizing the percentage of Blue^+^ cells of the adhesin mutants to WT. Experiments were repeated 3–8 times (**A–E**: *P<0.05, **P<0.01 and ***P<0.001 compared to WT; and **A–C**: ^+^P<0.05, ^++^P<0.01 and ^+++^P<0.001 compared to *ΔyopB*).

Three well-characterized *Yersinia* adhesins, Ail, Invasin and YadA [38]–[40], were assessed for their contribution to translocation of Yops into isolated splenocytes. Single, double and triple adhesin mutants were constructed in three different *Yptb* strains: IP2666, IP32953 and YPIII all of which are virulent in mouse infection models [3], [7], [41], yet differentially express *invasin* and *yadA* (Fig. S1A; [42], [43]). In order to measure Yop translocation all strains were engineered to express a reporter protein, ETEM. ETEM is a recombinant protein consisting of the N-terminus of *Yersinia* effector YopE, which contains the information necessary for translocation through the TTSS, fused with TEM, a β-lacatamse [44]. TEM cleaves the membrane permeable dye CCF4-AM, changing its fluorescence from green to blue. Using this reporter, TTSS-dependent translocation is measured by quantifying the number of cells that fluoresce blue [3], [45]. Splenocytes were infected with each ETEM-expressing strain at a multiplicity of infection (MOI) of 1∶1 loaded with CCF4-AM, and live cells were analyzed by flow cytometry for Yop translocation by measuring the number of Blue^+^ cells relative to WT (Fig. 1A–1C and Fig. S2A–B). A *ΔyopB* strain that is deficient for TTSS-dependent translocation served as a negative control [46]; as expected, it did not translocate Yops into splenocytes (Fig. 1A–1C and Fig. S2B).

Deleting all three adhesins, Ail, Invasin and YadA, dramatically reduced translocation into splenocytes by all three *Yptb* strains (Fig. 1A–1C and Fig. S2). However, individual strains relied on each adhesin to varying degrees. Notably, translocation by IP2666 was almost exclusively dependent on YadA and Ail, with YadA playing a predominant role; the role of Ail was apparent only in the absence of YadA (Fig. 1A and Fig. S2C). Complementing IP2666 *ΔailΔyadA* and *ΔailΔinvΔyadA* strains with either *ail* or *yadA* restored translocation to levels observed in the parental strain (Fig. S2C and Fig. S3A). In IP32953 and YPIII, translocation was primarily dependent on Invasin, with YadA playing a modest role in the absence of Invasin (Fig. 1B–1C). Complementation with a plasmid expressing Invasin restored the ability of *Δinv* and *ΔailΔinvΔyadA* strains to translocate Yops, in fact to levels greater than WT (Fig. S3B). This may be attributed to greater Invasin expression in the complemented strains (Fig. S1B). In summary, the combination of Ail, Invasin and YadA were necessary for Yop delivery into splenocytes in all three strains, but their individual contribution differed based on the strain background.

The translocation defect in a *Y. pestis Δail* mutant can be overcome by longer incubation with HEp2 cells [20]. This indicates that either productive receptor-adhesin interactions that compensate for Ail require more time for Yop delivery or that other adhesins capable of inducing translocation become expressed as the infection time lengthens. Therefore, we investigated if increasing the time or MOI of infection could overcome the translocation defect in the IP2666 adhesin mutants. Neither infecting splenocytes at an MOI of 1∶1 for 4 hours or at an MOI of 20∶1 for 1 hour led to a relative increase in the translocation efficiency of the *ΔailΔinvΔyadA* strain (Fig. 1D–1E). In fact, the percentage of Blue^+^ cells was unchanged for *ΔyadA* and *ΔinvΔyadA* strains but increased 5-fold following infection with a higher MOI of WT (Fig. S2E), leading to a net decrease in the relative number of cells translocated by strains lacking YadA compared to WT (Fig. 1A, 1D–1E). Collectively, these results indicate that the factor(s) responsible for the low levels of translocation in the *ΔailΔinvΔyadA* mutant are not enhanced by longer incubation times or higher MOIs. This suggests that the residual levels of translocation by this mutant could be due to adhesin-independent mechanisms [47].

A reduction in the overall number of cells injected with Yops should be reflected in a reduction in translocation into one or more specific splenic cell types. As expected, mutants where the overall levels of translocation were reduced compared to WT generally had a similar reduction in translocation into most cell types (Fig. S4A–C). For example, IP2666*ΔailΔyadA*, IP32953*ΔinvΔyadA* and YPIII*ΔinvΔyadA* strains translocated ETEM into fewer numbers of all cell types analyzed (Fig. S4B–C). A notable exception to this was that the IP32953*Δinv*, IP32953*ΔailΔinv*, YPIII*Δinv* and YPIII*ΔailΔinv* strains injected Yops into comparable numbers of Ly6G^+^ cells as did their isogenic WT strains. However, these strains translocated Yops into fewer F4/80^+^, CD11c^+^ and B220^+^ cells, which accounted for their overall lower levels of translocation (Fig. S4B–C).

**Figure 2:**
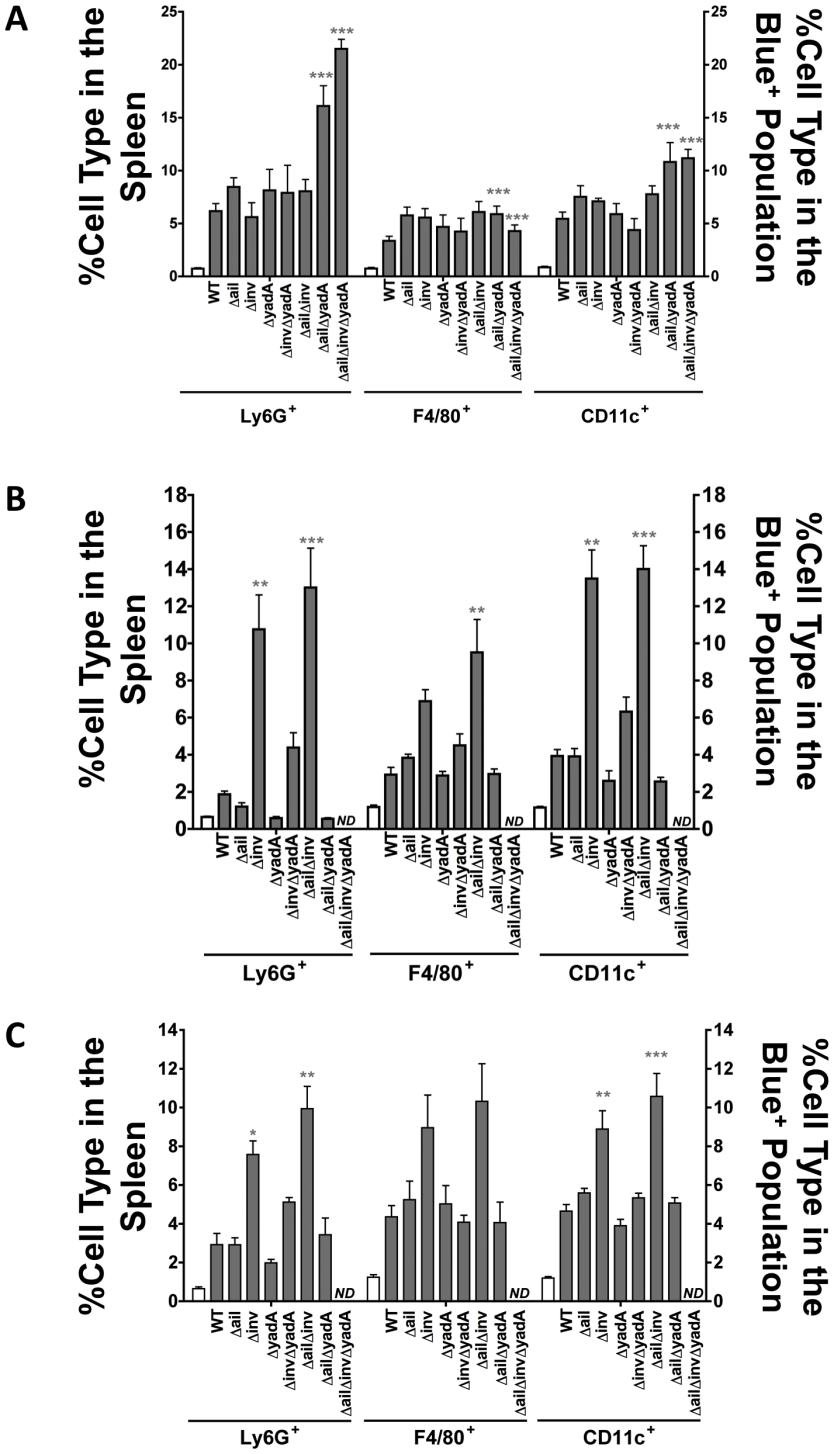
Adhesin mutants have variable, strain dependent effects on translocation into professional phagocytes. Splenocytes were infected with the indicated ETEM-expressing strains at an MOI of 1∶1 for (**A**) 1 h with IP2666, (**B**) 45 min with IP32953 strains or (**C**) 45 min with YPIII strains. Professional phagocytes were distinguished by cell-type surface marker staining using flow cytometry. The left Y-axis represents the percentage of each cell type in the spleen (white bars) while the right Y-axis represents the percentage of each cell type present in the Blue^+^ population (grey bars). Experiment was repeated 3–8 times (ND, not determined; *P<0.05, **P<0.01 and ***P<0.001 compared to WT).

Previous work has shown that neutrophils (Ly6G^+^), macrophages (F4/80^+^) and dendritic cells (CD11c^+^) are enriched in the cell population targeted for Yop delivery in both isolated splenocytes and during murine infection, compared to the abundance of these cell types in the whole splenocyte population [3]. Therefore, we examined whether the mutants translocated Yops into an altered spectrum of splenic cell types, as compared to WT. For this analysis, the percentage of a specific cell type in the Blue^+^ cell population for each mutant strain was compared to the percentage of that cell type in the Blue^+^ cell population from the isogenic WT strain (Fig. 2 and Fig. S5, grey bars). In strains where translocation levels were not significantly higher than *ΔyopB* strains (i.e. IP32953*ΔailΔinvΔyadA* and YPIII*ΔailΔinvΔyadA*, Fig. 1B–C), no cell type analysis was performed. Strikingly, most strains with dramatically reduced translocation levels (Fig. 1A–C) exhibited an even greater enrichment of professional phagocytes in the translocated cell population than was observed for WT (Fig. 2A–C). Furthermore, preferential targeting into Ly6G^+^, F4/80^+^ and CD11c^+^ cells was still observed by most adhesin mutant strains compared to the levels of these cells in the total splenocyte population (Fig. 2A–C gray bars versus white bar). The exceptions were the IP32953*ΔyadA* and *ΔailΔyadA* mutants, which did not translocate Yops into a greater percentage of Ly6G^+^ cells than found in the total splenocyte population (Fig. 2B). In addition, these two mutants consistently targeted fewer CD11c^+^ cells than did WT IP32953 (Fig. 2B), but this difference was not significant. Combined these results suggest that YadA promotes interactions with neutrophils and possibly dendritic cells for IP32953. With this exception, however, individual adhesins did not appear to play pivotal roles in directing Yop injection into professional phagocytes. Furthermore, phagocytes were generally over-enriched in the Blue^+^ population after infection with mutants that translocate Yops infrequently, suggesting that other factors contribute to the selective injection into these cells.

### IP2666 *ΔyadA* mutants bind to more B and T cell splenocytes

**Figure 3:**
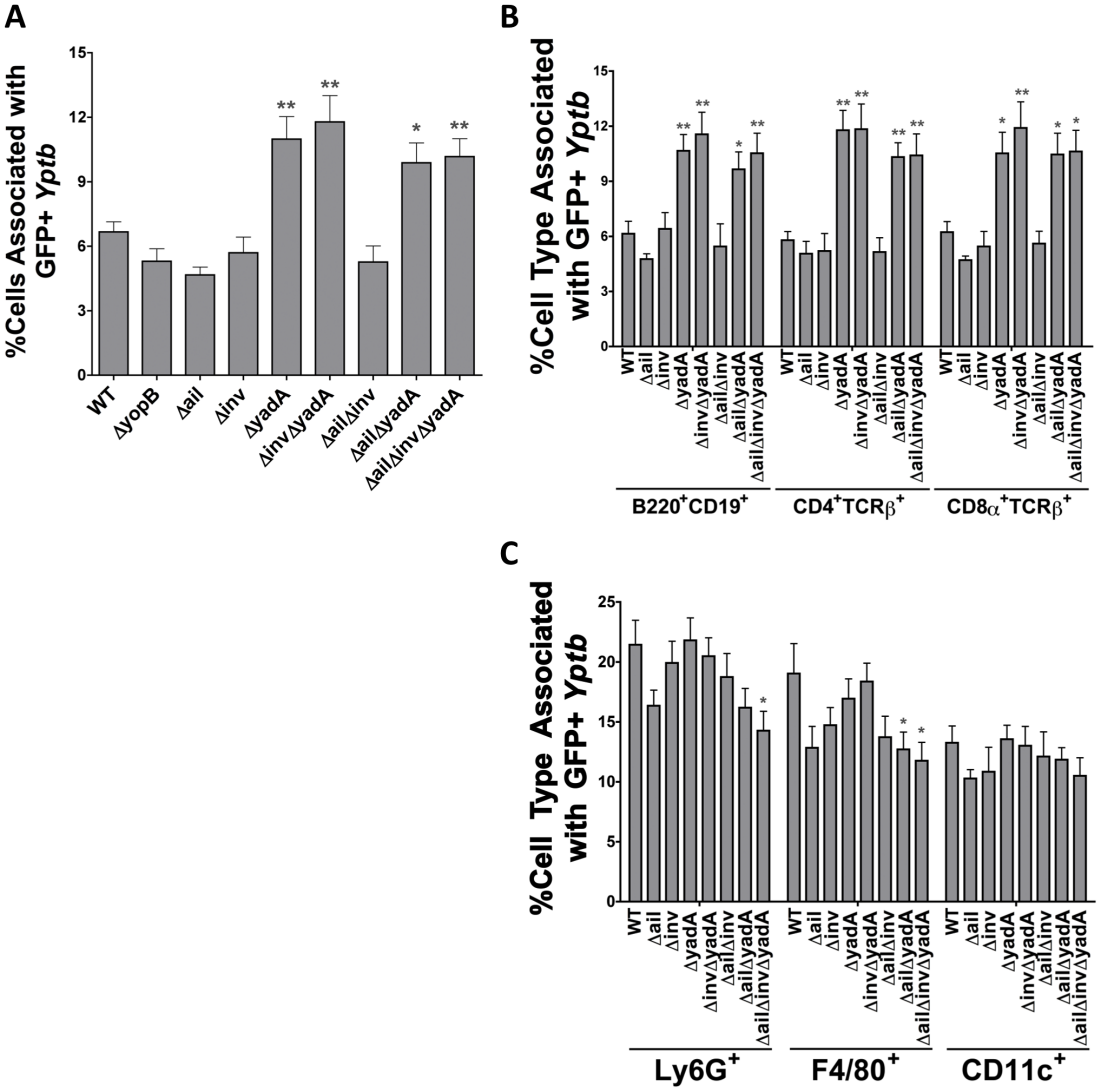
*ΔyadA* mutants associate more frequently with B and T cells than WT. (**A–C**) Splenocytes were infected at an MOI of 0.5∶1 with the indicated IP2666 GFP-expressing strains and cell types were distinguished by cell-type surface marker staining using flow cytometry. (**A**) The percentage of cells bound to GFP^+^ bacteria was determined by fluorescence intensity in the FITC channel of the total live splenocyte population. (**B and C**) The percentage of B and T cells (**B**) and phagocytes (**C**) bound by GFP^+^ bacteria. Experiment was repeated 5–8 times (*P<0.05, **P<0.01 and ***P<0.001 compared to WT).

Since binding to mammalian cells is a necessary step for Yop translocation [5], [10], [11] and as a number of adhesin mutants translocated Yops at low levels, these strains were evaluated for their ability to associate with splenocytes. GFP^+^ IP2666 *Yptb* adhesin mutants were incubated with splenocytes and specific cell types associated with bacteria were measured by flow cytometry. Surprisingly, the absence of YadA consistently led to an overall increase in *Yptb* association with splenocytes (Fig. 3A). Therefore, the defect in translocation of these strains (Fig. 1A) is not due to an inability to associate with splenocytes. Next, the percentage of each cell type associated with the adhesin mutants was compared with those associated with WT. Interestingly, strains lacking YadA associated with almost twice as many B220^+^CD19^+^, CD4^+^TCRβ^+^ and CD8α^+^TCRβ^+^ cells as compared to WT (Fig. 3B). Because CD4^+^TCRβ^+^, CD8α^+^TCRβ^+^ and B220^+^CD19^+^ cells make up a greater percentage of the total splenocyte population than Ly6G^+^, F4/80^+^ cells and CD11c^+^ cells (Fig. S6), this observation accounts for the higher overall association of *ΔyadA* strains with splenocytes. Fewer F4/80^+^ cells associated with *ΔailΔyadA* and *ΔailΔinvΔyadA* strains (Fig. 3C); however, since these cells are a minority of the overall splenocyte population (Fig. S6), this reduction was not great enough to offset the increased association observed with B and T cells (Fig. 3A–C). Combined, these results indicate that the defect in translocation is not a result of a defect in cell association. In fact, the *yadA* mutants bound to greater numbers of lymphocytes but this binding was not sufficient to drive translocation into a greater number of cells. These results are consistent with previous findings demonstrating that activation of host-cell signal transduction pathways by tight adhesin-receptor binding enhances translocation [11].

### Fewer host cells are translocated with Yops when mice are infected with a *Yptb ΔailΔinvΔyadA* mutant

**Figure 4:**
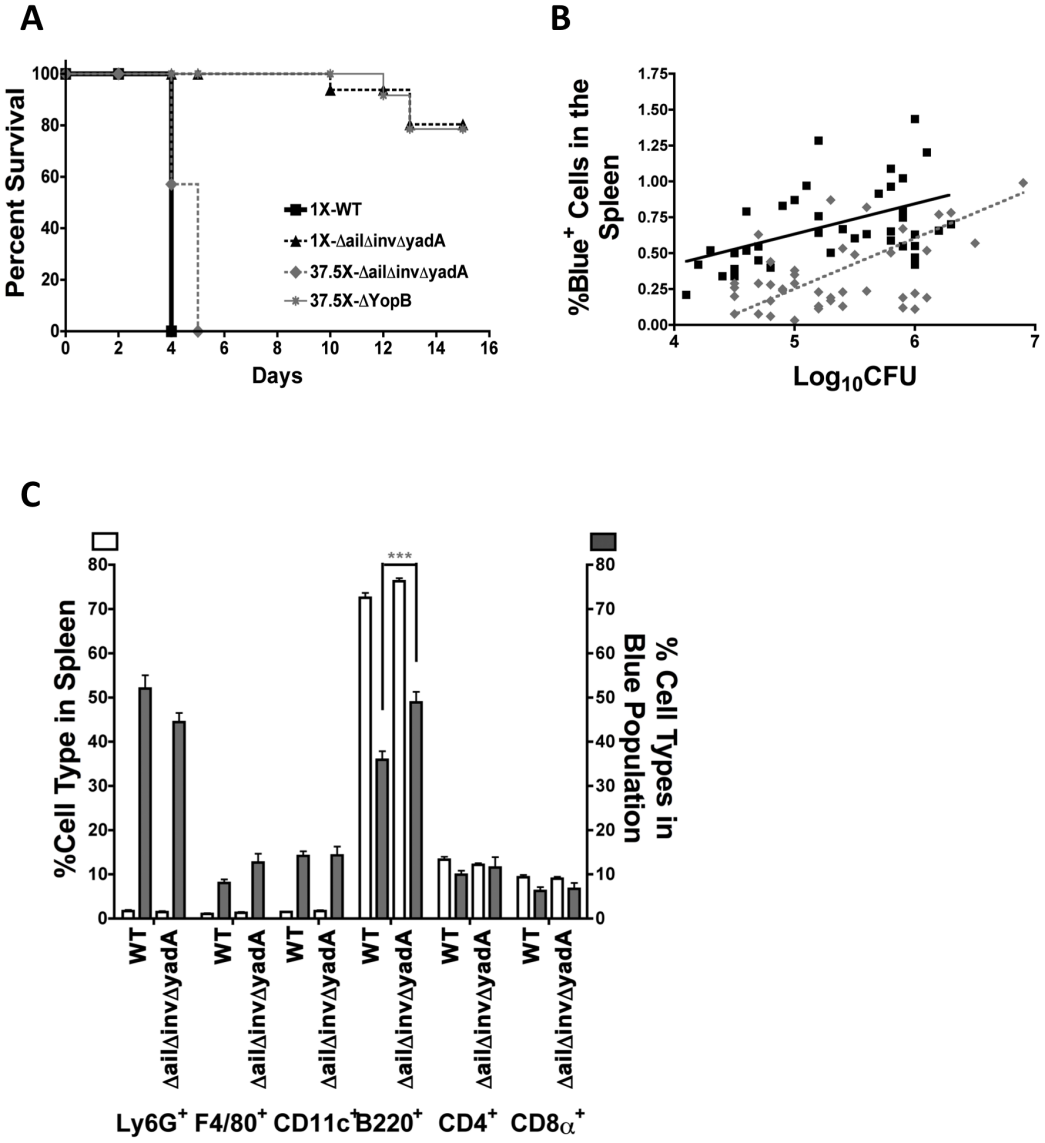
*ΔailΔinvΔyadA* is reduced in virulence and for Yop translocation during animal infections. Mice were infected IV with 800 CFU of IP2666 WT-ETEM (1X-WT) or *ΔailΔinvΔyadA-ETEM* (1X-*ΔailΔinvΔyadA*), or 30,000 CFU of *ΔailΔinvΔyadA-ETEM* (37.5X*ΔailΔinvΔyadA*) or *ΔyopB-ETEM* (37.5X-*ΔyopB*). (**A**) Animals were monitored for morbidity and mortality for a period of 15 days post infection and survival was plotted. (**B–C**) Four days post-infection, spleens were isolated and single cell suspensions were generated to enumerate CFU and percentage of Blue^+^ cells from mice infected with 800 CFU of WT-ETEM and 30,000 CFU of *ΔailΔinvΔyadA-ETEM*. (**B**) Black-filled squares with a solid black line represent values from mice infected with WT-ETEM and grey-filled diamonds with a dashed grey line represent values from mice infected with *ΔailΔinvΔyadA-ETEM*. Linear regression analysis determined that the percentage of Blue^+^ cells is significantly higher in animals infected with *WT-ETEM* than with *ΔailΔinvΔyadA-ETEM* (P = 0.0001). (**C**) The percentage of each cell type in the organ (white bars) compared with the percentage of each cell type present in the Blue^+^ population (grey bars) from spleens infected with WT-ETEM or *ΔailΔinvΔyadA-ETEM*. Only mice with greater than 4.8×10^4^ CFU were analyzed (***P<0.001).

Since IP2666 *ΔailΔinvΔyadA* failed to translocate Yops into isolated splenocytes, we postulated that these adhesins may contribute to efficiency of translocation during animal infection. However, in order to compare the translocation ability of these two strains during animal infection, they must colonize the tissue to similar levels. Due to the deficiency of *ΔailΔinvΔyadA* in translocating Yops to isolated splenocytes (Fig. 1A) and its sensitivity to killing by bovine serum (data not shown), we predicted that *ΔailΔinvΔyadA* would be attenuated in murine infection. To assess its relative virulence compared to WT, C57BL/6 mice were infected IV with 800 (1X) colony forming units (CFU) of WT-ETEM, 800 (1X) CFU of *ΔailΔinvΔyadA-ETEM*, 30,000 (37.5X) CFU of *ΔailΔinvΔyadA-ETEM* or 30,000 (37.5X) CFU of *ΔyopB-ETEM* and monitored for 15 days. All mice challenged with WT-ETEM succumbed to infection by the end of day 4, whereas 90% of mice challenged with 800 CFU of *ΔailΔinvΔyadA* survived, indicating that *ΔailΔinvΔyadA* was less virulent than WT (Fig. 4A). However, in contrast to *ΔyopB*, the *ΔailΔinvΔyadA-ETEM* strain caused a rapid, lethal infection when mice were challenged with 30,000 CFU. The observation that *ΔailΔinvΔyadA* was more virulent than *ΔyopB* suggested that the *ΔailΔinvΔyadA* mutant retained some ability to translocate Yops during animal infection.

We next investigated whether *ΔailΔinvΔyadA* delivered Yops into as many cells as WT when tissues were infected at comparable levels. To achieve comparable CFU in the spleen at 4 days post-infection, mice were infected with 800 CFU of WT-ETEM or 30,000 CFU of *ΔailΔinvΔyadA-ETEM*. Under these conditions, the onset of infection generally occurred in both groups of mice at the same time. To determine whether there was a difference in the levels of Yop translocation into cells, we compared the percentage of Blue^+^ cells versus the CFU recovered for the two strains (Fig. 4B). Strikingly, the *ΔailΔinvΔyadA* translocated Yops into significantly fewer cells than WT at comparable CFU at 4 days post-infection (Fig. 4B), as linear regression indicated a significant difference in the Y intercept (P<0.0001). This result demonstrates that one role of these adhesins in animal infections is to direct translocation of Yops into immune cells.

Next, the spectrum of host immune cells translocated into by the *ΔailΔinvΔyadA* mutant was compared to those cells translocated into by WT to determine whether adhesins direct Yops into specific cell types during animal infection. Interestingly, phagocyte enrichment in the Blue^+^ population was retained by the *ΔailΔinvΔyadA-ETEM* strain (Fig. 4C white bars versus gray bars). In addition, *ΔailΔinvΔyadA* injected Yops into relatively more B cells than WT (Fig. 4C). Taken together, these results revealed that although fewer cells are translocated by *ΔailΔinvΔyadA-ETEM* than by WT, professional phagocytes are still targeted for translocation. Therefore, adhesins contribute to the total numbers of cells injected with Yops, but other factors must contribute to the preferential translocation into professional phagocytes in spleens.

### Yop delivery to professional phagocytes is reduced in the absence of serum

**Figure 5:**
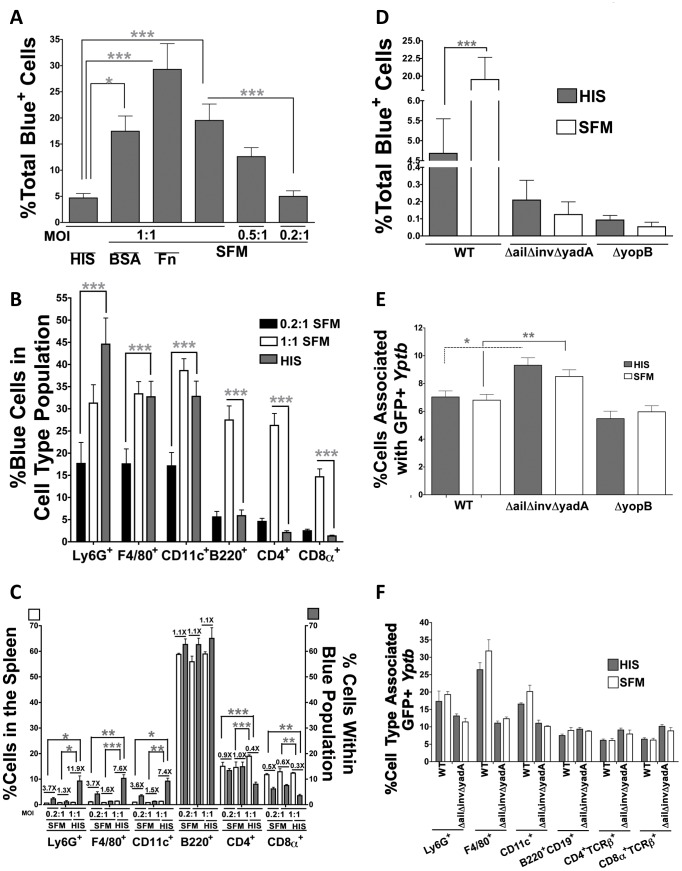
Serum directs Yop translocation into professional phagocytes. (**A–C**) Splenocytes in 5% HIS, Fn (40 µg/ml), BSA (5 mg/ml) or SFM were infected for 1 h with IP2666 WT-ETEM at an MOI of 0.2∶1, 0.5∶1 or 1∶1 and the percentage of Blue^+^ cells was determined by FACS. (**B–C**) Splenocytes infected for 1 h with IP2666 WT-ETEM at an MOI of 0.2∶1 or 1∶1 in SFM or an MOI of 1∶1 in HIS were analyzed for (**B**) the percentage of Blue^+^ cells in each cell type population or (**C**) the percentage of each cell type in the Blue^+^ population (gray bars) compared to the percentage of each cell type in the spleen (white bars). (**D**) Splenocytes were infected for 1 h at an MOI of 1∶1 with IP2666 WT-ETEM, *ΔailΔinvΔyadA-ETEM* or *ΔyopB-ETEM* and the percentage of Blue^+^ cells was determined. (**E–F**) Splenocytes were infected with the indicated IP2666 GFP^+^ strains at an MOI of 0.5∶1 and (**E**) the percentage of cells bound to GFP^+^ bacteria determined by fluorescence intensity in the FITC channel of the total splenocyte population, or (**F**) the percentage of specific cell types bound by GFP^+^ *Yptb*. Experiment was repeated 5–8 times (*P<0.05, **P<0.01 and ***P<0.001).

Opsonization of *Yersinia* by antibody or complement is sufficient to mediate binding and Yop delivery to phagocytes [5], [31]. Therefore, we tested whether bovine serum contributed to the total number or spectrum of cells of isolated splenocytes translocated into by *Yptb*. Splenocytes were infected at an MOI of 1∶1 with WT-ETEM in the presence of heat-inactivated Fetal Bovine Serum (HIS) or in serum free media (SFM) for 1 h. The bacteria and splenocytes survived equally well in the presence and absence of serum during the course of this assay (data not shown). Interestingly, infection in SFM led to a significant increase in the number of Blue^+^ cells (Fig. 5A), suggesting that a heat-resistant factor in serum restricts overall levels of Yop translocation.

To probe if serum contributes to the distribution of cell types targeted for Yop translocation, cell type distribution in the Blue^+^ population was compared between HIS versus SFM infections. Cell type distribution analysis was performed at comparable MOI (1∶1) for both conditions, as well as at an MOI of 0.2∶1 in SFM which results in similar overall numbers of Blue^+^ cells compared to infection in HIS at an MOI of 1∶1 (Fig. 5A). SFM infections at an MOI of 0.2∶1 led to significantly fewer translocated phagocytes, whereas at an MOI of 1∶1 a similar number of translocated phagocytes was obtained as compared to HIS infections at an MOI of 1∶1 (Fig. 5B). In contrast, at an MOI of 1∶1, significantly more B220^+^, CD4^+^ and CD8α^+^ cells were translocated into SFM than in HIS. Because CD4^+^, CD8α^+^ and B220^+^ cells make up a greater percentage of the total splenocyte population (Fig. S6), this observation accounts for the higher overall translocation levels in SFM at an MOI of 1∶1 (Fig. 5B). There were also changes in the distribution of cell types targeted for translocation among these conditions (Fig. 5C). Specifically, phagocytes were enriched and T cells were underrepresented in the Blue^+^ population of HIS-infected splenocytes compared to SFM (Fig. 5C). Therefore, bovine serum components curb overall levels of Yop translocation and contribute to the selective targeting of phagocytes by *Yptb*.

Since WT *Yptb* translocated Yops into more cells in SFM, we examined whether *ΔailΔinvΔyadA-ETEM* also translocated Yops more efficiently in SFM. However, *ΔailΔinvΔyadA-ETEM* remained defective for translocation in SFM (Fig. 5D), indicating that Ail, Invasin and/or YadA provide an essential interaction with host cells that drives translocation.

Both YadA and Ail bind to several extracellular proteins that facilitate contact with host cells [16], [25]. Two of these, fibronectin (Fn) and bovine serum albumin (BSA), were tested to determine if they modulated the number of and/or cell types translocated with Yops. A comparable number of splenocytes was translocated with Yops in SFM supplemented with Fn or BSA, indicating that neither Fn or BSA serve as the translocation-inhibiting factor in serum (Fig. 5A). Furthermore, infection in SFM supplemented with Fn or BSA led to reduced translocation of professional phagocytes (data not shown), as observed in SFM (Fig. 5B–5C) suggesting that these proteins do not alter the interaction of *Yptb* with phagocytes.

The increase in *Yptb* translocation efficiency in SFM could be driven by an increase in *Yptb* binding to host cells. To test this hypothesis, splenocytes were incubated with GFP^+^ WT, *ΔailΔinvΔyadA* or *ΔyopB* strains in HIS or in SFM at an MOI of 0.5∶1. The overall association of GFP-expressing *Yptb* strains with splenocytes was unchanged between HIS versus SFM (Fig. 5E). Furthermore, no difference in association with any of the cell types tested was observed in HIS versus SFM (Fig. 5F). Therefore, the changes in levels and cell types injected with Yops were not reflected in changes in association with splenocytes. Taken together, these results indicate that factors in serum mask interactions between *Yptb* and T cells that otherwise would result in translocation and enhance interactions between *Yptb* and professional phagocytes resulting in increased translocation.

### The virulence of *ΔailΔinvΔyadA* is restored in the absence of complement

**Figure 6:**
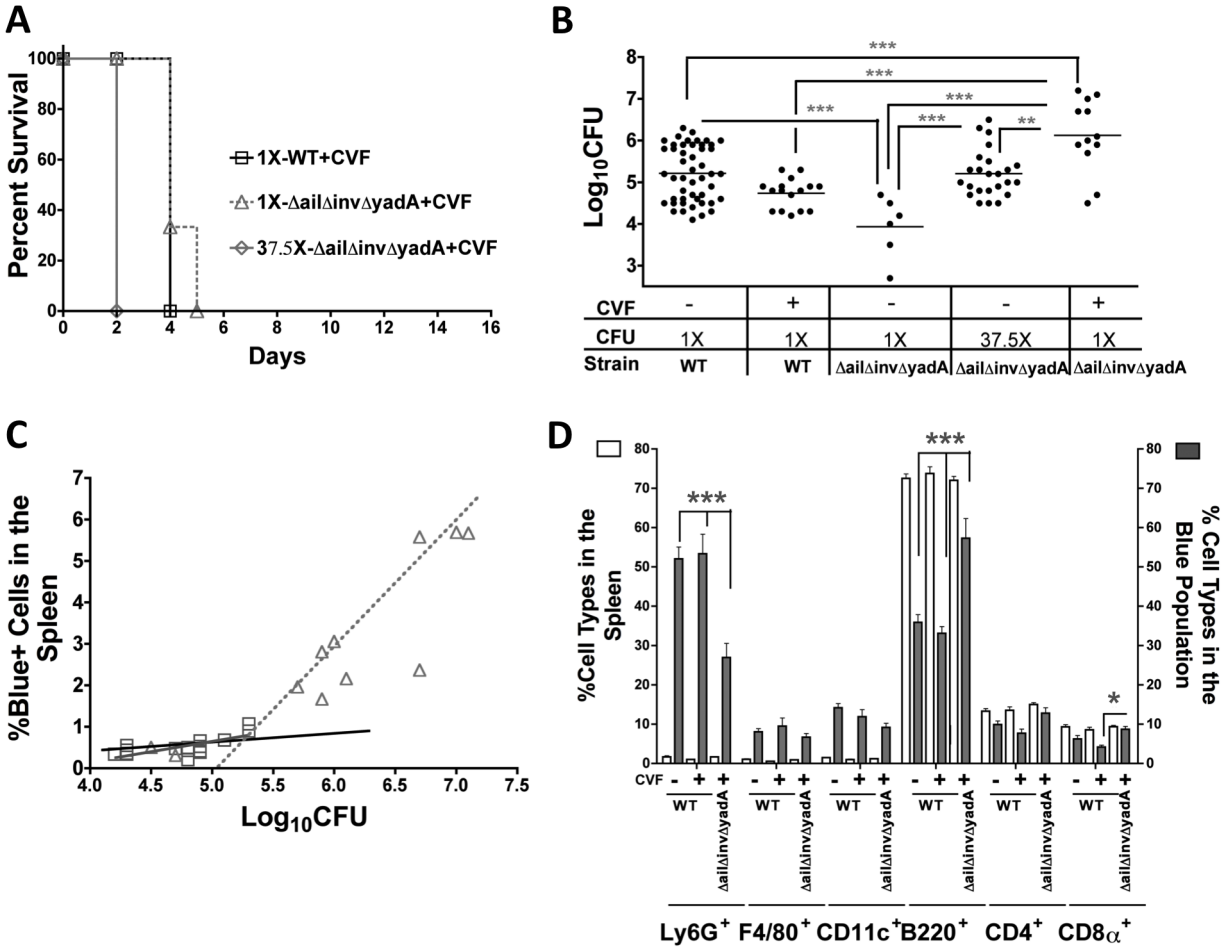
Complement depletion restores virulence and translocation of Yops by the *ΔailΔinvΔyadA* mutant. CVF-treated mice were infected IV with 800 CFU of IP2666 WT-ETEM (1X-WT), 800 CFU of *ΔailΔinvΔyadA-ETEM* (1X-*ΔailΔinvΔyadA*), or 30,000 CFU of *ΔailΔinvΔyadA-ETEM* (37.5X-*ΔailΔinvΔyadA*). (**A**) Animals were monitored for morbidity and mortality for a period of 15 days post infection and survival was plotted. (**B–D**) Four days post-infection, spleens were isolated to determine CFUs (**B**) and the percentage of Blue^+^ cells present in the organ compared to the log_10_CFU (**C**). (**B–C**) Each symbol represents data from one mouse; the bar in (**B**) represents the geometric mean. (**C**) The black line represents values from mice infected with 800 CFU WT-ETEM as shown in Fig. 4B, open-grey squares with grey line represents CVF-treated mice infected with 800 CFU WT-ETEM and open-grey triangles with dashed grey dotted line represent values from 800 CFU *ΔailΔinvΔyadA*-ETEM. Linear regression analysis determined that the percentage of Blue^+^ cells is the same in both WT infections regardless of CVF. In contrast, the CVF+1X-*ΔailΔinvΔyadA* injected Yops into significantly more cells than 1X-WT and CVF+1X-WT (with P<0.0001 for both). (**D**) The distribution of cell types found in the organ (white bars, left y-axis) versus the distribution of cell types found in the Blue^+^ population (gray bars, right y-axis) for each infection condition was compared. (**B and D**) (**P<0.01 and ***P<0.001). Data from mice infected with 800 CFU of WT-ETEM and 30,000 CFU of *ΔailΔinvΔyadA* in panels **B–D** are from the same mice analyzed in Fig. 4B–C.

While heat inactivation of serum eliminates the ability of complement to form MAC and lyse bacteria [48], complement components still present in serum could bind adhesins and direct *Yptb* to specific receptors on professional phagocytes. To determine whether complement components play a role in controlling infection and/or directing *Yptb* to translocate Yops into specific cell types, we depleted complement by injecting cobra venom factor (CVF) intraperitoneally into mice [49]. CVF is a complement-activating protein in cobra venom that forms an active convertase complex [50] that is resistant to inactivation by host complement regulatory proteins. As a result of its constitutive activity, the CVF-convertase depletes serum and tissues of complement components. Following complement depletion, mice were infected IV with 800 (1X) CFU of WT-ETEM, 800 (1X) CFU of *ΔailΔinvΔyadA-ETEM*, or 30,000 (37.5X) CFU of *ΔailΔinvΔyadA-ETEM* and monitored for 15 days. Notably, CVF treatment restored the virulence defect of the *ΔailΔinvΔyadA* strain but had no effect on the virulence of WT (Fig. 4A and 6A). Consistent with this observation, CVF treatment greatly enhanced the number of *ΔailΔinvΔyadA* bacteria present in spleens but not WT (Fig. 6B). This was also consistent with our observation that in the absence of complement, the *ΔailΔinvΔyadA* infected mice generally displayed signs of illness, such as a hunched appearance and slower walk, a few hours before mice infected with WT. Combined, these results indicate that complement controls growth of the *ΔailΔinvΔyadA* strain but not of WT. Therefore Ail, Invasin and/or YadA must be counteracting the action of complement during mouse infection.

To investigate whether complement-depletion altered the number of cells injected with Yops, CVF-treated mice were infected IV with 800 CFU of WT-ETEM or 800 CFU of *ΔailΔinvΔyadA-ETEM*. Four days post infection, the number of injected cells under these two infection conditions was also compared to non-complement depleted mice infected with 800 CFU of WT-ETEM (Fig. 4B and solid black line in Fig. 6C). No difference in the levels of Blue^+^ cells was observed in mice infected with WT-ETEM, with or without complement depletion, when similar CFUs were recovered from the spleen (Fig. 6C, solid black line versus solid grey line). In contrast, once the *ΔailΔinvΔyadA-ETEM* strain reached levels of 5.5×10^5^ in the absence of complement, it delivered Yops to significantly more cells than did WT (Fig. 6C dashed lines, versus solid lines). However, significantly more *ΔailΔinvΔyadA* bacteria were recovered in these tissues, which could explain the increased number of cells injected with Yops (Fig. 6B). Therefore, we compared the number of cells injected by *ΔailΔinvΔyadA* and WT when comparable numbers of bacteria were recovered (Fig. S7A–B). Again, under these conditions, the *ΔailΔinvΔyadA* strain in CVF-treated mice injected more cells than did WT. In summary, this analysis illustrates that in the absence of complement, *ΔailΔinvΔyadA* translocates Yops into more cells than WT, indicating that complement components affect both bacterial survival and Yop translocation. Furthermore, the significant increase in growth and efficiency of translocation of the *ΔailΔinvΔyadA* mutant under complement-depleted conditions suggest that this mutant behaves quite differently than WT and/or is in a distinctly different microenvironment.

We next examined whether complement depletion affected the spectrum of cells translocated with Yops by either WT or *ΔailΔinvΔyadA*. No difference was observed in the cell types targeted for Yop translocation by WT-ETEM in the presence or absence of complement (Fig. 6D). Interestingly, in the absence of complement, the *ΔailΔinvΔyadA-ETEM* strain exhibited significantly decreased translocation into Ly6G^+^ cells and increased translocation into B220^+^ cells, as compared to WT and WT+CVF (Fig. 6D and Fig. S7C). These results combined with those in Fig. 4C suggest that complement components direct translocation of Yops from *ΔailΔinvΔyadA* to Ly6G^+^ cells.

## Discussion

In order to mount a successful infection, *Yersinia* must counteract a multitude of host immune responses that are enlisted to control an invading pathogen. One essential mechanism *Yersinia spp.* use to thwart immune responses is to deliver Yop effector proteins into host cells via a TTSS [8]. These Yops quickly disrupt signal transduction pathways that are normally geared to respond to invading threats and thus allow *Yersinia* to successfully colonize and persist in the host [8], [9]. This work demonstrates that both bacterial adhesins and host factors contribute to the efficiency of translocation and the specificity of immune cell types translocated with Yops. Furthermore, adhesins contribute to resistance of host complement.

**Figure 7:**
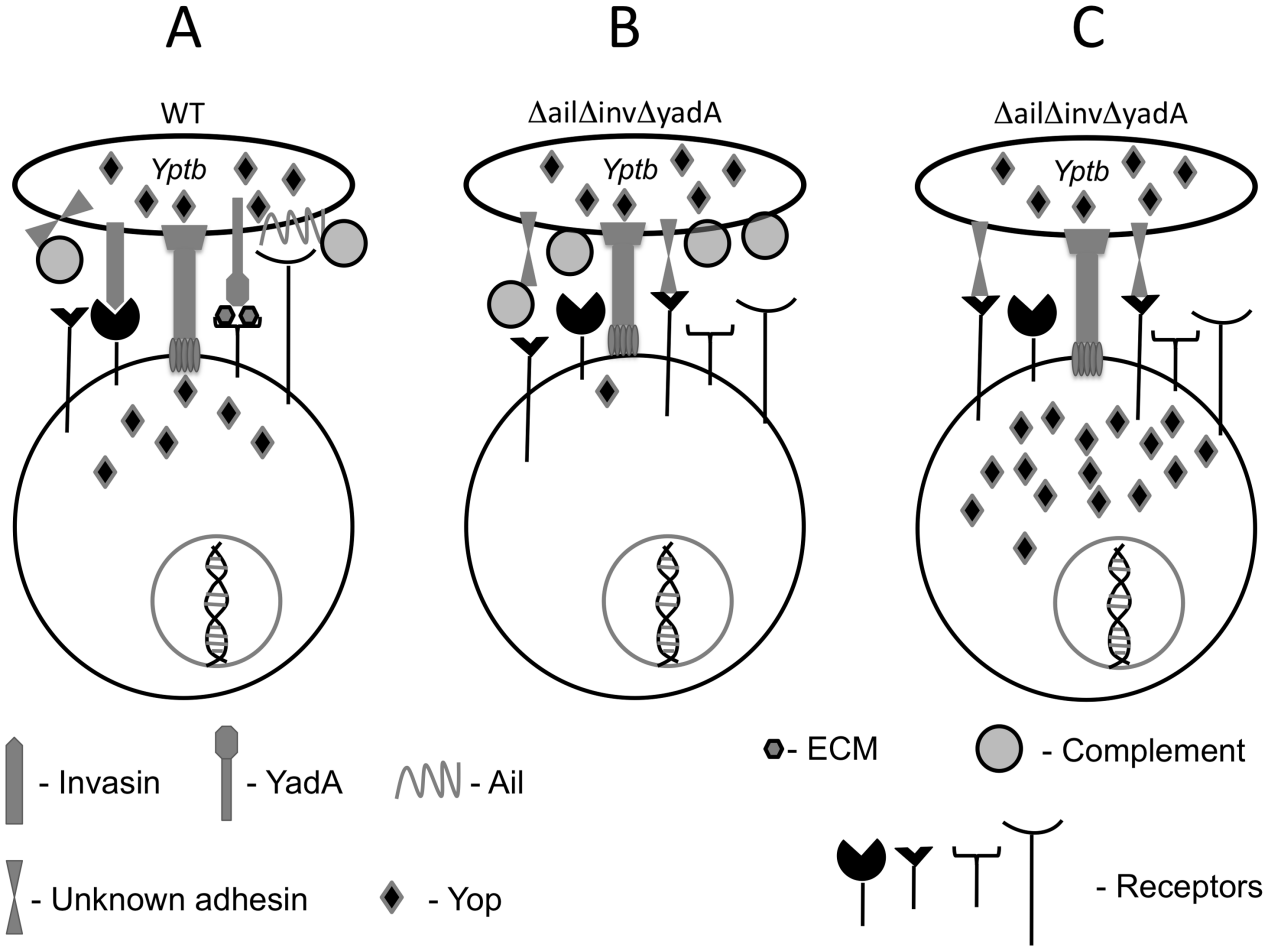
A model of factors that drive Yop translocation during mouse infection. (**A**) During infection, *Yptb* expresses adhesins Ail, Invasin and YadA, which mediate binding to host-cell receptors directly or indirectly via ECM components or complement, leading to TTSS engagement and Yop delivery into host cells. (**B**) In a *ΔailΔinvΔyadA* mutant, another unknown *Yptb* adhesin becomes accessible and mediates Yop delivery. Factors such as complement dampen proper engagement of the unknown adhesin with host cells leading to fewer Yop translocated cells. (**C**) In the absence of complement, an unknown adhesin becomes accessible in the *ΔailΔinvΔyadA* mutant and mediates TTSS engagement and Yop delivery into host cells.

In mouse infection and in isolated splenocytes, a *ΔailΔinvΔyadA* strain translocated Yops into significantly fewer cells than WT. These results indicate that the three adhesins are important for the vast majority of translocation that occurs during infection. Thus, one critical feature of *Yersinia* adhesins is to direct translocation of Yops in infected tissues (Fig. 7A). However, the low yet detectable level of translocation by the *ΔailΔinvΔyadA* mutant *in vivo* indicates that other factors can also drive translocation in the absence of these adhesins (Fig. 7B). These factors may include additional adhesins and/or host factors. For example, growth conditions can dictate the expression of major virulence factors [43], [51]. Growth in human plasma, which may mimic a bloodstream environment, positively regulates levels of YadA but negatively regulates pH 6 antigen in *Yptb* [51]. Some bacterial factors capable of driving translocation may be expressed poorly or function inefficiently during *in vitro* culture conditions, but may contribute to translocation during animal infection. Such potential factors include, *ifp* and *invC*, which are expressed by *Yptb* in the mouse intestinal tract but not *in vitro* [52]. On the other hand, the low level of Yop translocation by *ΔailΔinvΔyadA in vivo* may be due to host factors that direct translocation by bridging the *Yersinia* outer surface to receptors on phagocytes. In fact, our results show that host factors also contributed to Yop delivery in isolated splenocytes and during mouse infection.

In cell culture systems, serum promotes TTSS-mediated secretion by *Pseudomonas*, *Shigella*, *Salmonella* and *Yersinia* [32], [53]–[55]. In addition, BSA, a major component of serum, activates TTSS-mediated secretion by *Y. enterocolitica* and it has been proposed that serum components such as BSA provide signals for TTSS activation by *Yersinia* [32]. Thus, one hypothesis is that *Yersinia* exposed to serum or BSA would translocate effectors at higher levels because it is already primed to secrete Yops. Surprisingly, bovine serum restricted overall translocation levels into isolated splenocytes, but enhanced translocation specifically into neutrophils, indicating that factors in serum modulate the efficiency and specificity of Yop translocation.

To further investigate the role of serum components in directing Yop translocation during animal infection, we used CVF to deplete complement. Depletion of complement revealed several important facets of infection. Notably, we demonstrate that the adhesins Ail, Invasin and YadA function to ameliorate the actions of complement in animal infection, as shown by the difference in survival of the *ΔailΔinvΔyadA* strain in complement depleted versus non-depleted mice. Moreover, complement-depletion enhanced Yop translocation by *ΔailΔinvΔyadA* but not by WT. To our knowledge, this is the first report of a role for adhesins in counteracting the activities of complement during murine infection by *Yersinia*. Complement contributes to a rapid immune response during infection by promoting inflammation, mediating bacteriolysis by MAC formation, and providing oponsins for phagocytosis [33], [34]. A *Y. pestis Δail* strain is sensitive to the killing action of complement from a wide variety of sources except mice [48], and protection afforded by Ail is evident in rats where *Y. pestis Δail* is completely attenuated [56], [57]. Combined, these observations suggest that the attenuation of the *Y. pestis Δail* strain in rats is due to killing by complement, although this was not demonstrated directly. Mouse complement lacks bactericidal activity due to a nonfunctional C5 convertase and does not kill a *Y. pestis Δail* strain [36], [37], [48]. However, other functions of complement, such as opsonization and promotion of inflammation, are retained. In mice a *Y. pestis Δail* mutant exhibits a delayed time to death [56], suggesting that the mutant is partially sensitive to aspects of murine host defenses. Our data is consistent with the hypothesis that these activities may be mediated by complement.

Why does depleting complement affect the growth of *ΔailΔinvΔyadA* but not WT *Yptb*? Complement components may coat the mutant, rendering it susceptible to opsonophagocytosis (Fig. 7). This, combined with inefficient Yop translocation into neutrophils, may contribute to its elimination. In the absence of complement, innate immune cells may no longer efficiently bind the mutant, allowing for its enhanced growth. WT may be exempt from being opsonophagocytosed as it efficiently delivers Yops into innate immune cells, a number of which block phagocytosis [5]. In addition, WT may inhibit generation of pro-inflammatory chemoattractants C3a and C5a [21], [22], whereas these chemoattractants may be produced at higher levels during infection with *ΔailΔinvΔyadA*, resulting in increased neutrophil recruitment. It has been proposed that Ail stifles neutrophil recruitment by complement during *Y. pestis* infection [57]. Ail, however, does not inhibit neutrophil recruitment to *Yptb* because neutrophils surround WT *Yptb* microcolonies [58], [59]. Complement may also elicit release of neutrophil extracellular traps (NETs) [60], which may be involved in controlling *Yptb* infection. NETs are extracellular fibers composed of granule and nuclear constituents that disarm and kill extracellular bacterial pathogens [61]. By trapping bacteria, NET release limits systemic dissemination [60], [62]. The complement-inhibitory action of Ail and/or YadA may inhibit NET release during WT infection, whereas a *ΔailΔinvΔyadA* mutant would be unable to inhibit NET release and thus becomes efficiently trapped and killed by innate immune cells. In the absence of complement, NETs are not released [60], which may account for the growth of *ΔailΔinvΔyadA* as the mutant may no longer be trapped and killed by NETs.

Complement-depletion also enhanced Yop translocation and altered the spectrum of cells targeted for translocation by the *ΔailΔinvΔyadA* strain but not by WT. In cell culture, opsonization of *Yersinia* by complement or antibody is sufficient to mediate binding and Yop delivery to macrophages and neutrophils [5], [31], but in mouse infection, complement had no effect on translocation by WT. We speculate that with a full repertoire of adhesins, WT *Yptb* may be fully equipped to bind cells and deliver Yops regardless of complement (Fig. 7A). In the absence of Ail, Invasin and YadA, another *Yptb* adhesin and/or host factor may promote translocation (Fig. 7A–B); however, complement can now contain the infection and dampen or hinder the *Yptb*-host cell interactions required for efficient translocation (Fig. 7B). In the absence of complement, this factor can mediate translocation by *ΔailΔinvΔyadA* (Fig. 7C) and direct injection of Yops into a different subset of immune cells. The observation that *ΔailΔinvΔyadA* is fully virulent in the absence of complement, despite the fact that it targets an altered spectrum of cells, could be due to several possibilities. First, while neutrophils are not targeted to the same degree as by WT, they are still targeted. This level of targeting combined with a presumable reduction in opsonophagocytosis in the absence of complement may be sufficient to permit growth of the mutant. Second, the absence of both complement and adhesins may permit the bacteria to reside within a privileged niche that it does not normally occupy. For instance, these bacteria may be internalized more frequently by a subset of macrophages than is normally observed for *Yptb* [63], [64] and thus may be protected from neutrophils. Finally, in the absence of complement, WT may be vulnerable to other immune responses due to its expression of adhesins. For example, YadA enhances binding to NETs and thus its expression may increase the susceptibility of WT to bactericidal factors released in NETs [60], [65]. In the absence of both YadA and complement, *Yersinia* would not become trapped as readily in these NETs. These scenarios are consistent with the idea that depending on the tissue environment, adhesins can either facilitate bacterial survival or elimination by host [65].

In summary, translocation and targeting of Yops into host cells during infection is a multi-factorial process whereby multiple *Yersinia* adhesins and host factors act together. Bacterial adhesins contribute to the total number of cells targeted for translocation, and *Yersinia* employs host complement to help drive Yop translocation into specific cell types. Future work will be aimed at identifying the complement component(s) required for this function, investigating the relative contribution of each of these adhesins to serum resistance and Yop translocation during infection, and identifying other adhesins that facilitate translocation during animal infection.

## Materials and Methods

### Strains and bacterial culture conditions

Strains (Table S1), primers (Table S2), plasmids, and strain construction are described in Methods S1.

### Mice infection and complement depletion

Mice were infected as described [7] with the following modifications. 7–8 week old C57BL/6 (NCI) mice were infected IV with 100 µl of the strain and dose indicated in the legends for Figures 4 and 6. Mice were either sacrificed on day 4 or monitored for up to 15 days until they displayed significant signs of illness. CVF (Quidel Corporation) was used to deplete complement as described [49]. Briefly, C57BL/6 mice were injected intraperitoneally with two 5 Unit doses of CVF 4 h apart. 24 hours later, mice were infected IV with *Yptb*.

### Ethics statement

This study was carried out in accordance with the recommendations in the Guide for Care and Use of Laboratory Animals of the National Institutes of Health. The Institutional Animal Care and Use Committee of Tufts University approved all animal procedures. Our approved protocol number is B2012-54. All efforts were made to minimize suffering; animals were monitored following infection and were euthanized upon exhibiting substantial signs of morbidity by CO_2_ asphyxiation followed by cervical dislocation.

### CCF4-AM conversion assays

Spleens were harvested aseptically, treated with collagenase D (Roche), and a cell suspension was generated as described [3]. Unless indicated in the figure legends, cell suspensions were resuspended in RPMI supplemented with 5% heat-inactivated fetal bovine serum (HIS). Single cell suspensions were incubated for 30 minutes in the dark in media containing 1 µg/ml CCF4-AM (Invitrogen), 1.5 mM probenecid (Sigma) and 100 µg/ml gentamicin. 100 µl of cells were aliquoted into a 96-well plate and incubated with 50 µl of FACS buffer (PBS+1% HIS) containing a 1∶200 dilution of Mouse BD Fc Block (BD) for 10 minutes at 4°C. Cells were incubated in 50 µl of FACS buffer containing fluorescent antibodies Ly6G-PE-Cy7, B220-PeCy5, CD19-Cy7, CD4-PECy5, CD8α-PECy5, TCRβ-Cy7 (BD Pharmingen), F4/80-PE-Cy5 and/or CD11c-PECy5 (eBioscience) at dilutions of 1∶75 for 30 minutes at 4°C, washed twice in FACS buffer, centrifuged at 340× g, resuspended in 200 µl in FACS buffer and analyzed on an LSRII (Becton Dickson) FACS machine. 5×10^4^–4×10^5^ cells were acquired per sample and data was analyzed using FlowJo v4.3 software. For strains that injected Yops at levels less than 30% of those observed for WT, 4×10^5^ live cells were collected for analysis. Otherwise, 5×10^4^ live cells were analyzed. Uninfected cells that were not incubated with CCF4-AM and/or antibodies were used as negative controls.

### Assay of *Yptb* adherence to splenocyte suspension

Splenocyte adherence assays were performed as described previously [3] with the following modifications. Single cell suspensions were resuspended in RPMI without serum (SFM) or media supplemented with 5% HIS as indicated in the figure legends. Cells were infected with the indicated GFP-expressing strains at an MOI of 0.5∶1 for 20 minutes at 37°C. Cells were labeled with antibodies and analyzed by flow cytometry.

### Statistical analysis

For all figures, except Figures 3, 4B, 6C and S7B–C, data was graphed and statistical analysis was performed using GraphPad PRISM software by applying One-Way Anova with Tukey's Multiple Comparison Test. For Figure 3 statistical analysis was performed by applying One-Way Anova with Dunnett's Multiple Comparison Test. For Figures 4B and 6C linear regression analysis was performed using SAS9.2 analytical software in collaboration with the Tufts Clinical and Translational Science Institute (CTSI). For Figure S7B–C analysis was performed by applying Student's t test.

## Supporting Information

Figure S1 **Different** ***Yptb*** **strains express varying levels of Invasin and YadA.** (**A**) *Yptb* strains were cultured in 2XYT supplemented with 5 mM CaCl_2_ and grown at 26°C for 2 h followed by 2 h at 37°C. Bacteria were washed and incubated for 1 h at 37°C in RPMI media supplemented with 5% HIS. Bacteria were lysed in SDS sample buffer and lysates were analyzed by western blot analysis using antibodies specific for Ail, Invasin or YadA. (**B**) *Yptb* strains were cultured in 2XYT supplemented with 5 mM CaCl_2_ and grown at 26°C for 2 h followed by 2 h at 37°C. IPTG was added at a concentration of 0.2 mM when cultures were switched to 37°C. Bacteria were lysed in SDS sample buffer and lysates were analyzed by western blot analysis using antibody specific for Invasin. (**A–B**) Blot images are a representative of three independent experiments.
(TIF)

Figure S2 ***ΔailΔinvΔyadA*** **mutants translocate Yops poorly into isolated splenocytes as compared to WT.** (**A**) The percentage of viable cells in the splenocyte suspension was determined by PI staining. Panel 1 shows the percentage of PI^+^ cells in the total splenocyte population; Panel 2 depicts where the PI^+^ cells fall within the FSC vs SSC plot and the gate G1 where we exclude 80% of the PI^+^ cells; Panel 3 shows the G1 on the total splenocyte population analyzed by FSC vs SSC; and Panel 4 shows the number of PI^+^ cells present in the G1 gate of the FSC and SSC analysis (panel 3). (**B–E**) Splenocytes were infected with the indicated ETEM-expressing strains and the percentage of Blue^+^ cells was determined by flow cytometry. (**B**) Green^+^ and Blue/Green^+^ (hereafter called Blue^+^) gates from live cells from G1 in (**A**) panel 3, were gated based on uninfected, unstained live cells, uninfected splenocytes plus CCF4-AM, and splenocytes infected with *ΔyopB-ETEM* (designated *ΔyopB* in **B**). These gates were compared to splenocytes infected with WT-ETEM or *ΔailΔinvΔyadA*-ETEM. (**B**) Splenocytes were infected for 1 h with an MOI of 1∶1 for 1 h with IP2666 strains, 45 min with YPIII strains or 45 min with IP32953 strains. **(C–E**) Splenocytes were infected with IP2666 strains (**C**) for 1 h at an MOI of 1∶1, (**D**) for 4 h at an MOI of 1∶1, or (**E**) for 1 h at an MOI of 20∶1. Experiment was repeated 3–5 times (* P<0.05 and *** P<0.001 compared to WT).
(TIF)

Figure S3 **Complementing with ail, inv or yadA restored Yop translocation into isolated splenocytes by** ***ΔailΔinvΔyadA*** **adhesin mutants.** (**A–B**) Splenocytes were infected with the indicated ETEM-expressing strains at an MOI of 1∶1 for 1 h with IP2666 (**A**) or at an MOI of 1∶1 for 45 minutes with YPIII strains (**B**). CCF4 conversion from green to blue was measured by flow cytometry and the relative percentage of Blue^+^ cells was determined by setting WT to 1 and normalizing the percentage of Blue^+^ cells of the adhesin mutants to WT. Experiment was repeated 3–5 times (* P<0.05, ** P<0.01 and *** P<0.001 compared to WT).
(TIF)

Figure S4 **Translocation deficient mutants exhibit reduced translocation to different splenic cell types.** Splenocytes were infected with the indicated ETEM-expressing strains at an MOI of 1∶1 for (**A**) 1 h with IP2666 strains, (**B**) 45 min with IP32953 strains or (**C**) 45 min with YPIII strains. The percentage of Blue^+^ cells in each cell-type population as defined by the markers indicated on the X-axis was determined. (ND, not determined; * P<0.05, ** P<0.01 and *** P<0.001 compared to WT).
(TIF)

Figure S5 **YadA mutants have variable effects on translocation into B-cells that are strain dependent.** Splenocytes were infected with the indicated ETEM-expressing strain for 1 h with IP2666 (**A**), 45 min with IP32953 (**B**) or 45 min with YPIII (**C**) at an MOI of 1∶1. B-cells (**A and B**) and T-cells (**A**) were distinguished by flow cytometry. The left Y-axis represents the percentage of each cell type in the spleen (white bars) while the right Y-axis represents the percentage of each cell type present in the Blue^+^ population (grey bars). The experiment was repeated 3–5 times (ND, not determined; ** P<0.01 and *** P<0.001 compared to WT).
(TIF)

Figure S6 **Cell Type Distribution in Spleens.** Splenocyte suspensions were incubated with the indicated cell-type specific antibodies to distinguish different cells in the spleen by flow cytometry. Graph represents the percentage of each cell type in the entire organ. The experiment was repeated 5 times.
(TIF)

Figure S7 ***ΔailΔinvΔyadA*** **translocates Yops into more cells in complement-depleted mice when similar levels of CFUs as WT are achieved in the spleens.** (**A**) is the same as Fig. 6B with grey circles to indicate the subset of mice infected with WT and CVF-treated mice infected with 1x-*ΔailΔinvΔyadA* that were analyzed in (**B–C**). No statistically significant difference in the CFU recovered from the circled mice was observed (Student's t test). (**B**) The percentage of Blue^+^ cells in the subset of mice circled in (**A**) was significantly different between the two groups of mice (Student's t test). (**C**) The distribution of cell types found within the organ (white bars, left y-axis) vs the distribution of cell types found in the Blue^+^ population (gray bars, right y-axis) for each infection condition was compared. (* P<0.05, ** P<0.01 and *** P<0.001).
(TIF)

Table S1 **Strains and Plasmids.** List of strains and plasmids constructed and/or used in this study. The *Yptb* strain from which the *Yptb* insert was cloned into pCVD442 is indicated in parenthesis.
(DOCX)

Table S2 **List of Primers.** List of primers used in this study to generate pCVD442 plasmids with *Yptb* inserts.
(DOCX)

Methods S1 Supporting Materials and Methods.
(DOCX)
