# Supplementary figures and images for: Adhesins and Host Serum Factors Drive Yop Translocation by Yersinia into Professional Phagocytes during Animal Infection
Source: PLoS Pathog. 2013 Jun 20;9(6):e1003415. doi: 10.1371/journal.ppat.1003415 (PMC3688556; doi:10.1371/journal.ppat.1003415)

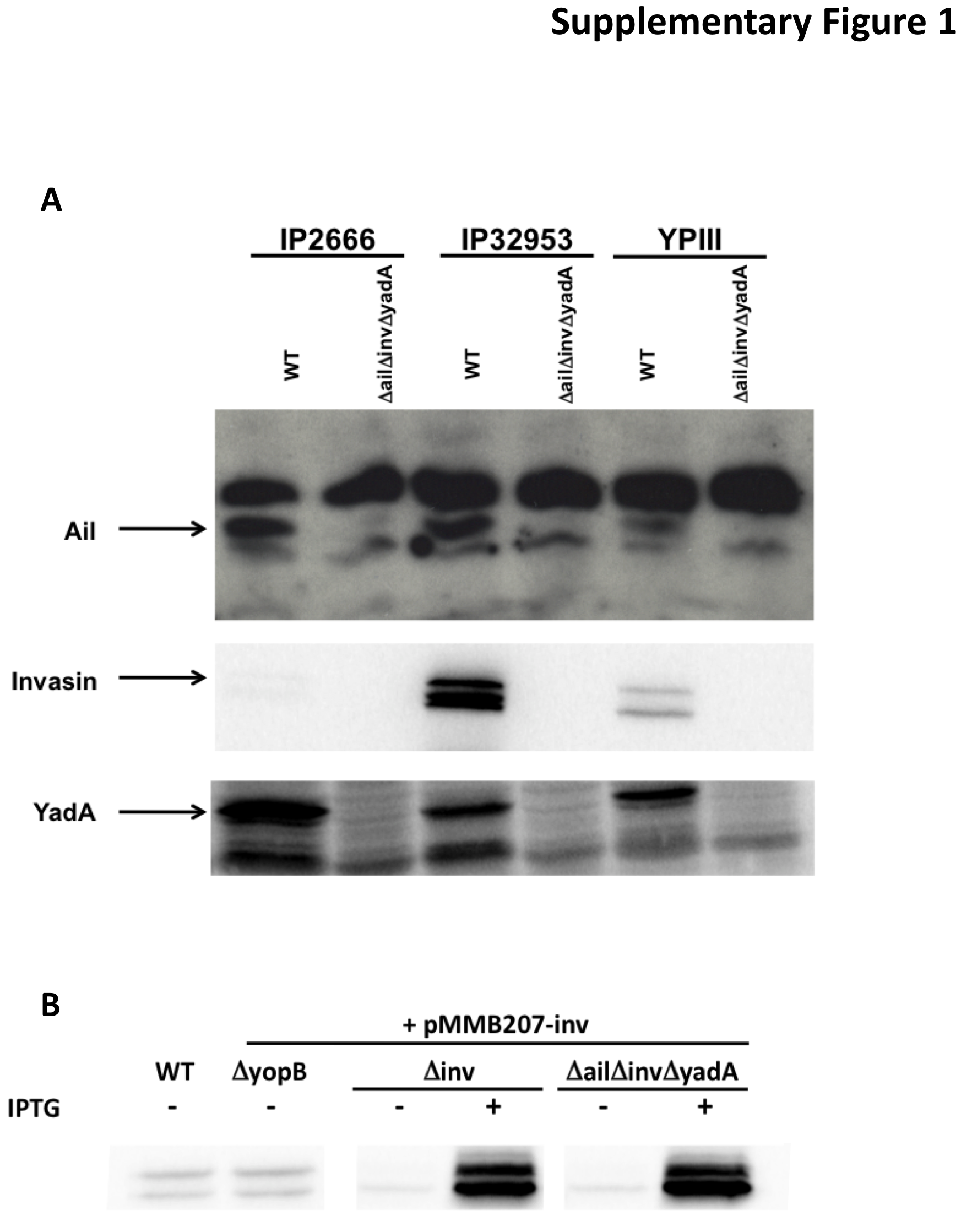

Supplement: Figure S1 — Different Yptb strains express varying levels of Invasin and YadA. (A) Yptb strains were cultured in 2XYT supplemented with 5 mM CaCl2 and grown at 26°C for 2 h followed by 2 h at 37°C. Bacteria were washed and incubated for 1 h at 37°C in RPMI media supplemented with 5% HIS. Bacteria were lysed in SDS sample buffer and lysates were analyzed by western blot analysis using antibodies specific for Ail, Invasin or YadA. (B) Yptb strains were cultured in 2XYT supplemented with 5 mM CaCl2 and grown at 26°C for 2 h followed by 2 h at 37°C. IPTG was added at a concentration of 0.2 mM when cultures were switched to 37°C. Bacteria were lysed in SDS sample buffer and lysates were analyzed by western blot analysis using antibody specific for Invasin. (A–B) Blot images are a representative of three independent experiments. (TIF) [file ppat.1003415.s001.tif]

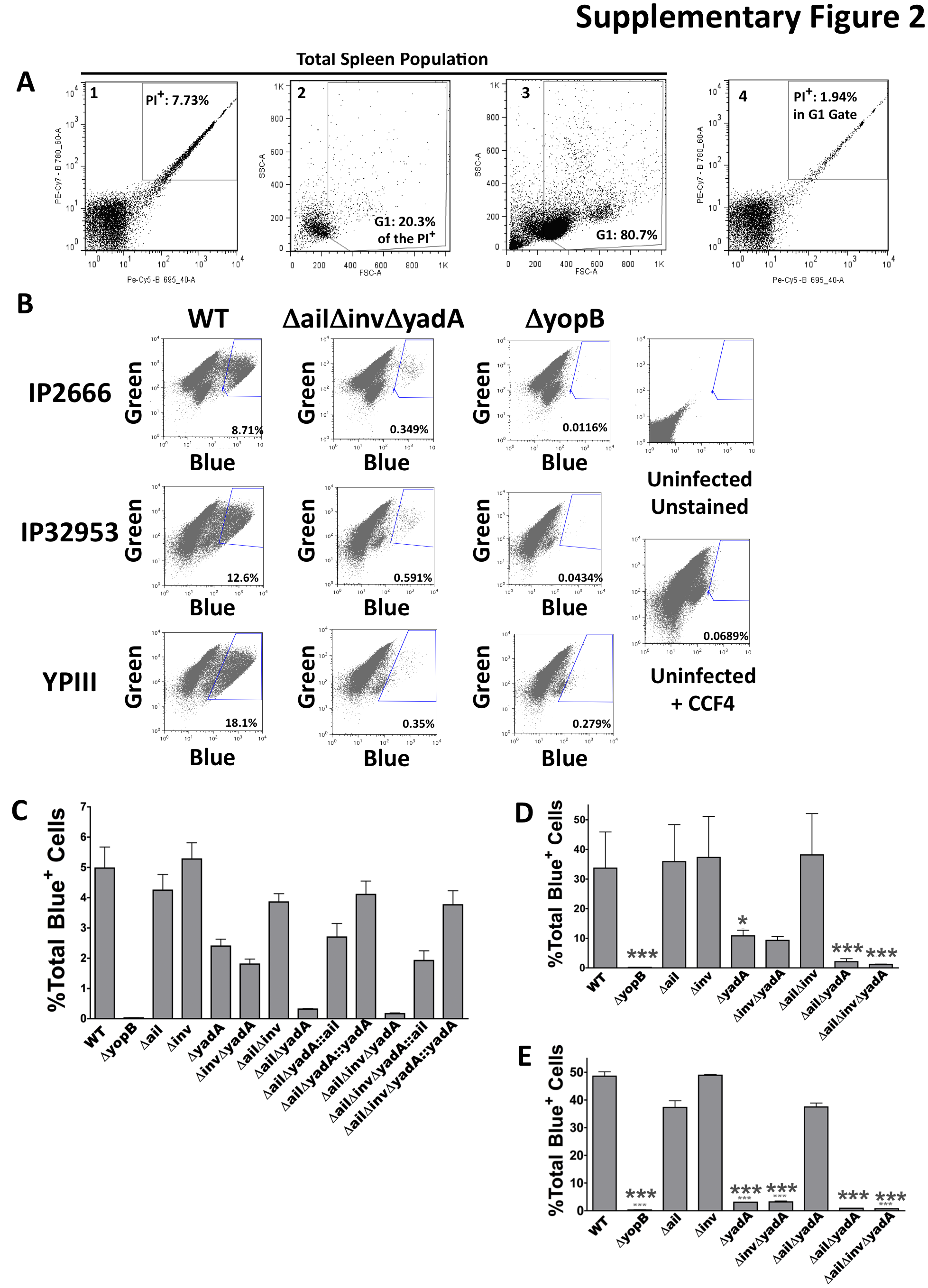

Supplement: Figure S2 — ΔailΔinvΔyadA mutants translocate Yops poorly into isolated splenocytes as compared to WT. (A) The percentage of viable cells in the splenocyte suspension was determined by PI staining. Panel 1 shows the percentage of PI+ cells in the total splenocyte population; Panel 2 depicts where the PI+ cells fall within the FSC vs SSC plot and the gate G1 where we exclude 80% of the PI+ cells; Panel 3 shows the G1 on the total splenocyte population analyzed by FSC vs SSC; and Panel 4 shows the number of PI+ cells present in the G1 gate of the FSC and SSC analysis (panel 3). (B–E) Splenocytes were infected with the indicated ETEM-expressing strains and the percentage of Blue+ cells was determined by flow cytometry. (B) Green+ and Blue/Green+ (hereafter called Blue+) gates from live cells from G1 in (A) panel 3, were gated based on uninfected, unstained live cells, uninfected splenocytes plus CCF4-AM, and splenocytes infected with ΔyopB-ETEM (designated ΔyopB in B). These gates were compared to splenocytes infected with WT-ETEM or ΔailΔinvΔyadA-ETEM. (B) Splenocytes were infected for 1 h with an MOI of 1∶1 for 1 h with IP2666 strains, 45 min with YPIII strains or 45 min with IP32953 strains. (C–E) Splenocytes were infected with IP2666 strains (C) for 1 h at an MOI of 1∶1, (D) for 4 h at an MOI of 1∶1, or (E) for 1 h at an MOI of 20∶1. Experiment was repeated 3–5 times (* P<0.05 and *** P<0.001 compared to WT). (TIF) [file ppat.1003415.s002.tif]

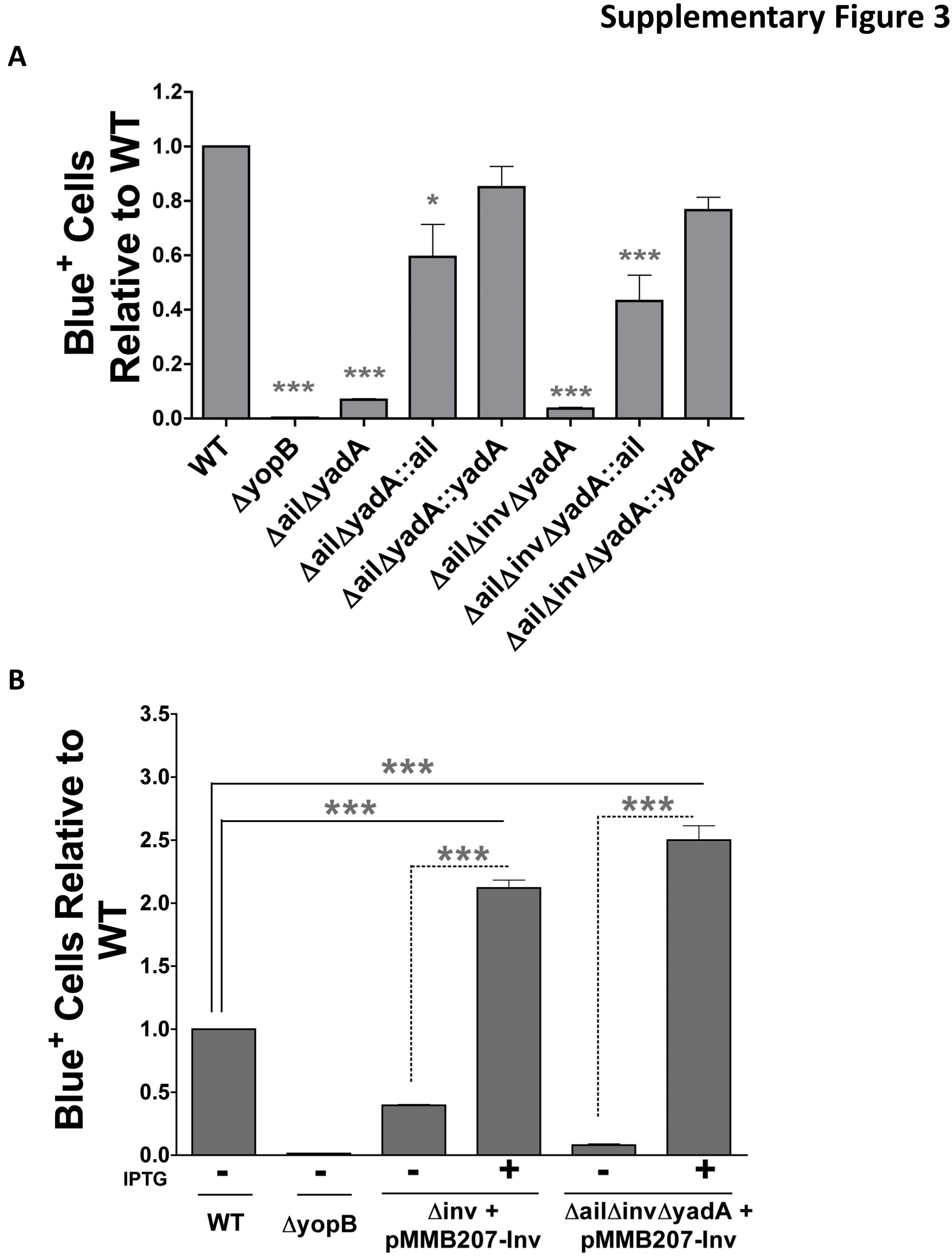

Supplement: Figure S3 — Complementing with ail, inv or yadA restored Yop translocation into isolated splenocytes by ΔailΔinvΔyadA adhesin mutants. (A–B) Splenocytes were infected with the indicated ETEM-expressing strains at an MOI of 1∶1 for 1 h with IP2666 (A) or at an MOI of 1∶1 for 45 minutes with YPIII strains (B). CCF4 conversion from green to blue was measured by flow cytometry and the relative percentage of Blue+ cells was determined by setting WT to 1 and normalizing the percentage of Blue+ cells of the adhesin mutants to WT. Experiment was repeated 3–5 times (* P<0.05, ** P<0.01 and *** P<0.001 compared to WT). (TIF) [file ppat.1003415.s003.tif]

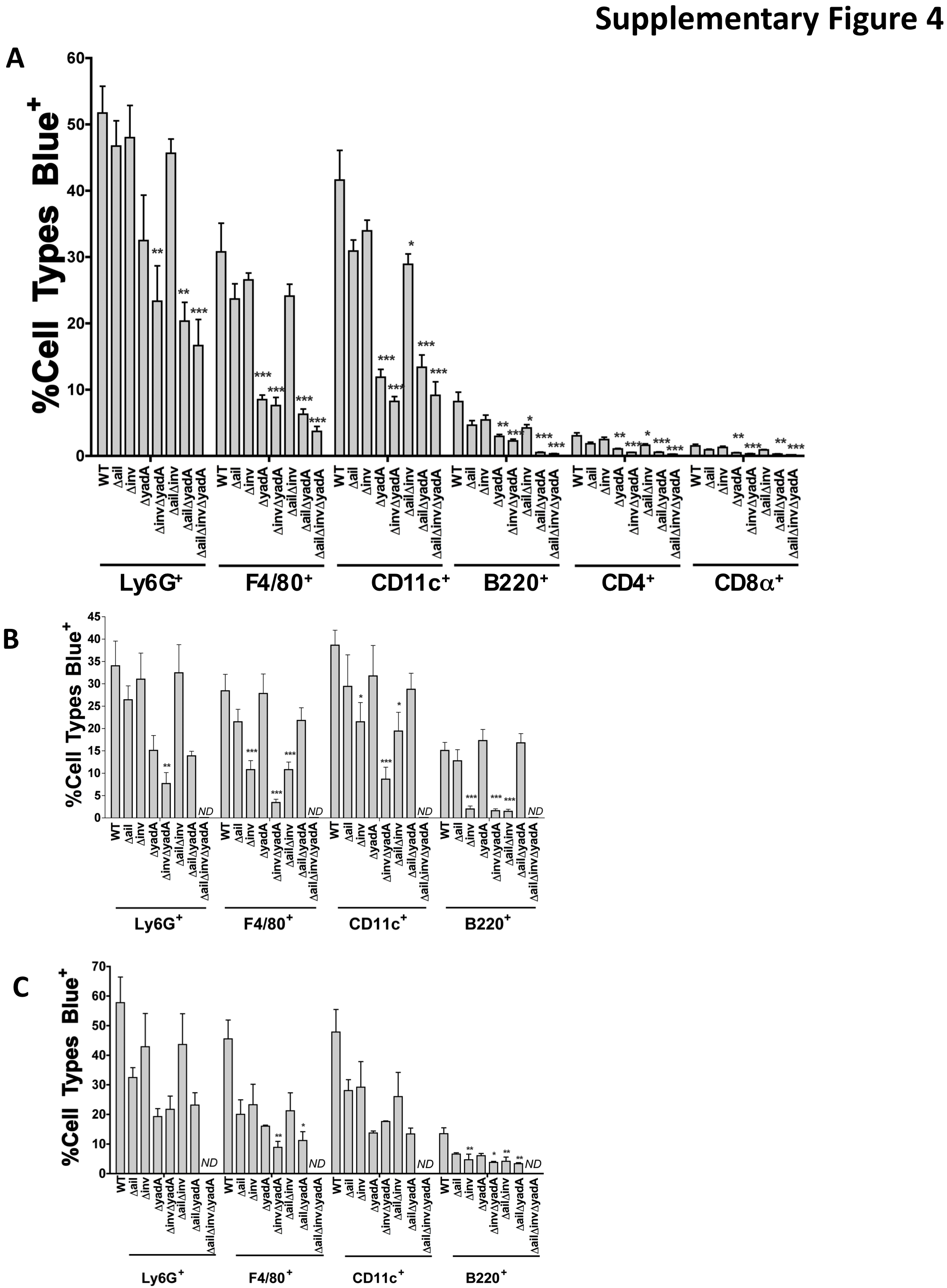

Supplement: Figure S4 — Translocation deficient mutants exhibit reduced translocation to different splenic cell types. Splenocytes were infected with the indicated ETEM-expressing strains at an MOI of 1∶1 for (A) 1 h with IP2666 strains, (B) 45 min with IP32953 strains or (C) 45 min with YPIII strains. The percentage of Blue+ cells in each cell-type population as defined by the markers indicated on the X-axis was determined. (ND, not determined; * P<0.05, ** P<0.01 and *** P<0.001 compared to WT). (TIF) [file ppat.1003415.s004.tif]

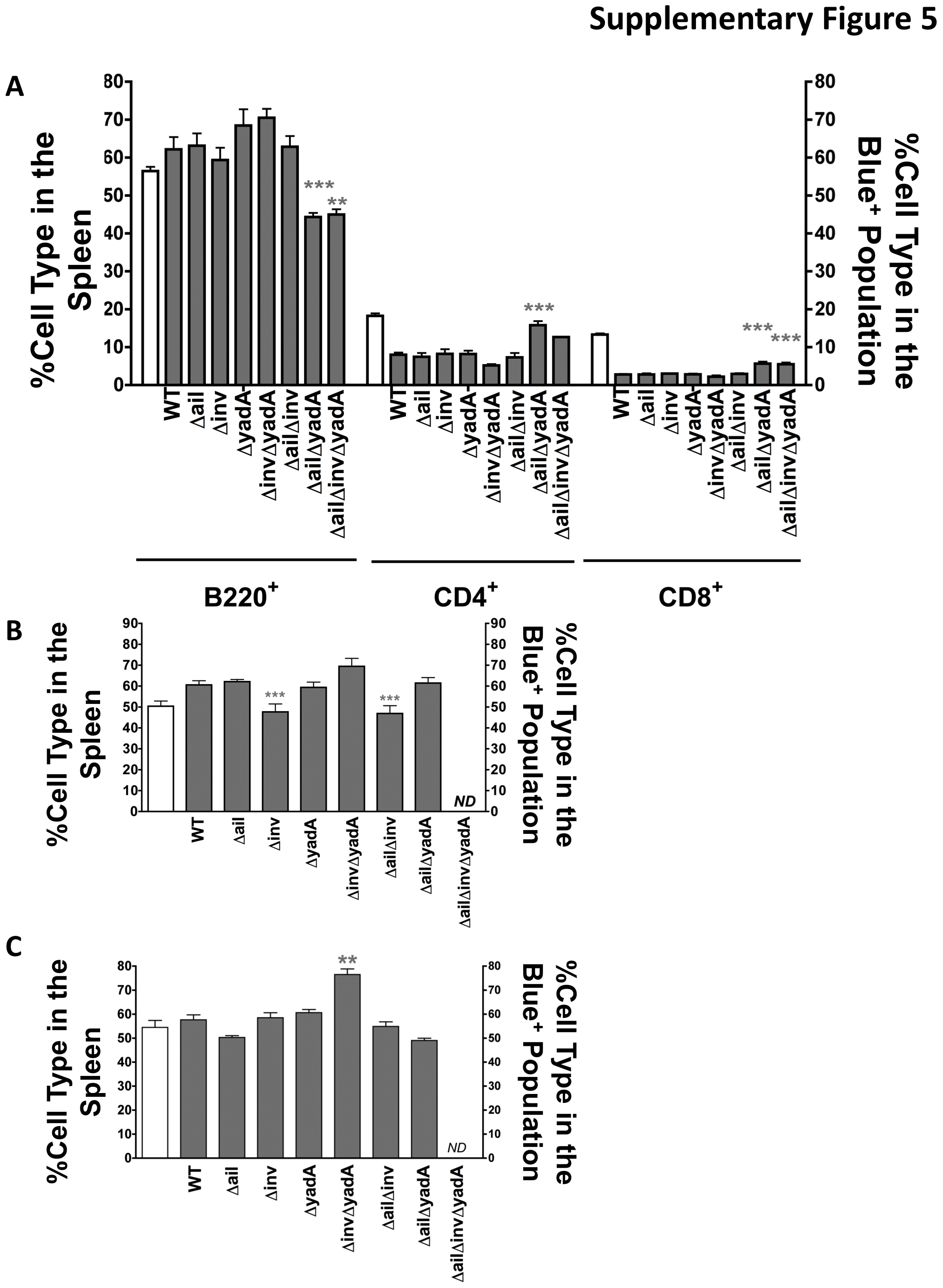

Supplement: Figure S5 — YadA mutants have variable effects on translocation into B-cells that are strain dependent. Splenocytes were infected with the indicated ETEM-expressing strain for 1 h with IP2666 (A), 45 min with IP32953 (B) or 45 min with YPIII (C) at an MOI of 1∶1. B-cells (A and B) and T-cells (A) were distinguished by flow cytometry. The left Y-axis represents the percentage of each cell type in the spleen (white bars) while the right Y-axis represents the percentage of each cell type present in the Blue+ population (grey bars). The experiment was repeated 3–5 times (ND, not determined; ** P<0.01 and *** P<0.001 compared to WT). (TIF) [file ppat.1003415.s005.tif]

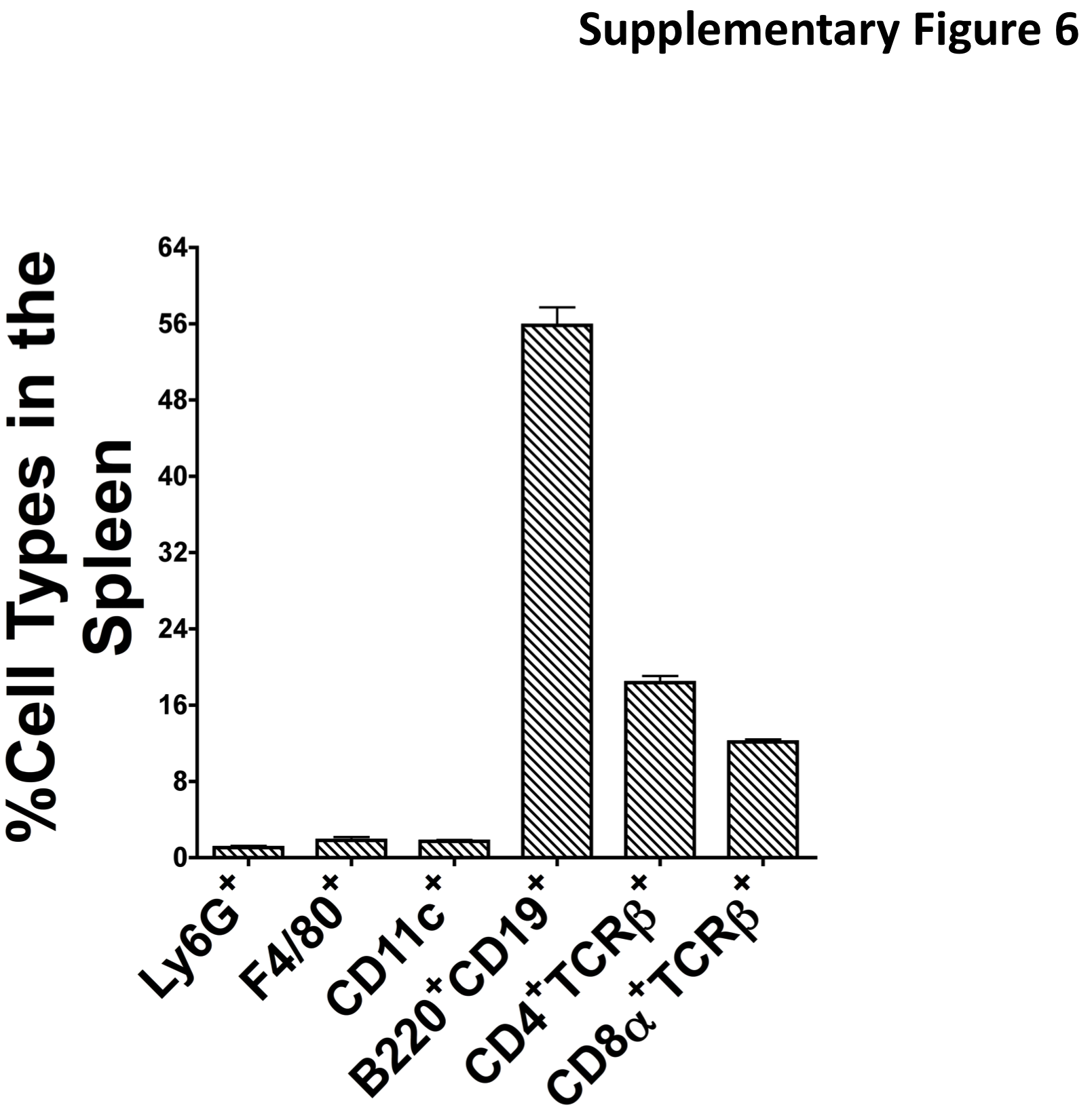

Supplement: Figure S6 — Cell Type Distribution in Spleens. Splenocyte suspensions were incubated with the indicated cell-type specific antibodies to distinguish different cells in the spleen by flow cytometry. Graph represents the percentage of each cell type in the entire organ. The experiment was repeated 5 times. (TIF) [file ppat.1003415.s006.tif]

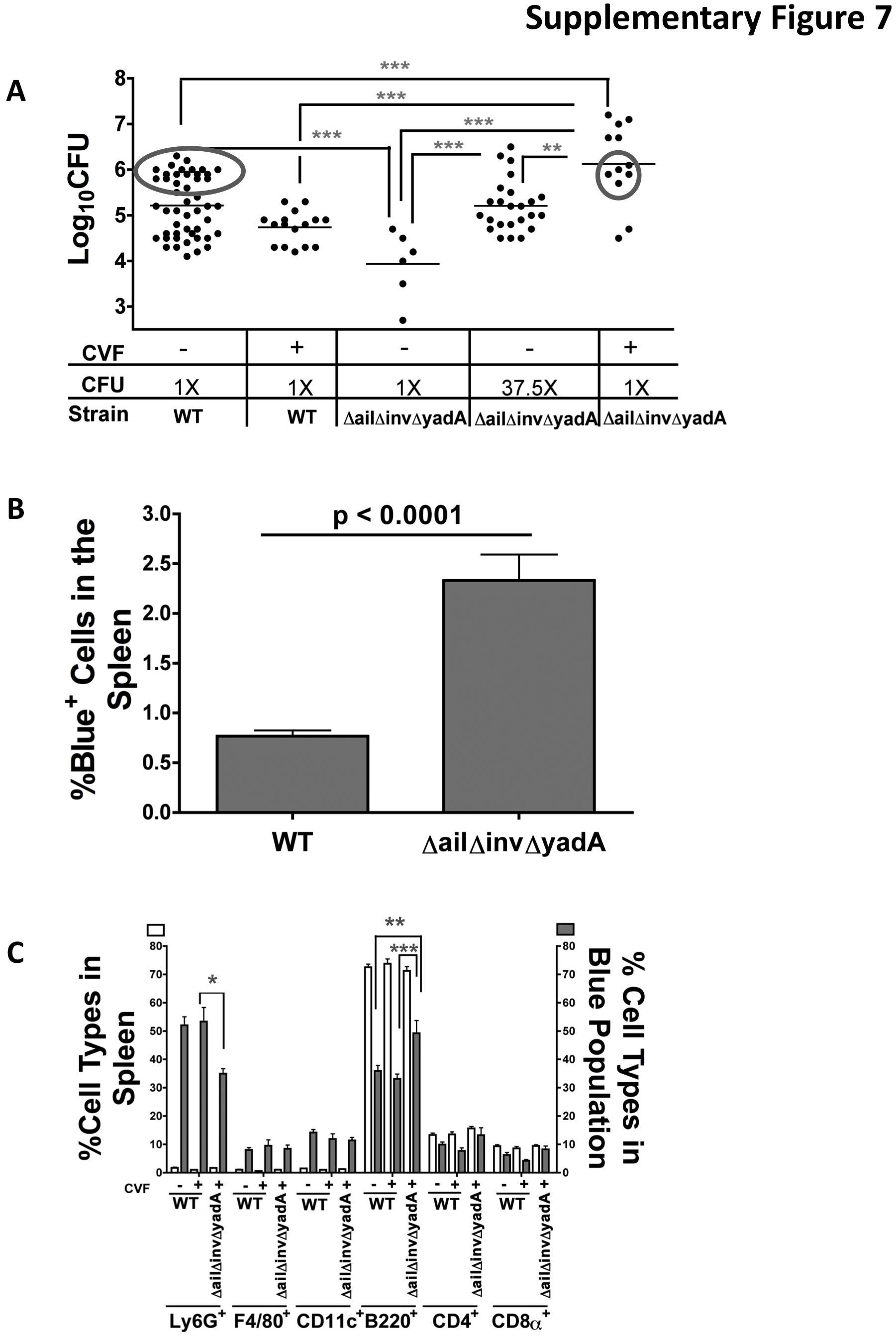

Supplement: Figure S7 — ΔailΔinvΔyadA translocates Yops into more cells in complement-depleted mice when similar levels of CFUs as WT are achieved in the spleens. (A) is the same as Fig. 6B with grey circles to indicate the subset of mice infected with WT and CVF-treated mice infected with 1x-ΔailΔinvΔyadA that were analyzed in (B–C). No statistically significant difference in the CFU recovered from the circled mice was observed (Student's t test). (B) The percentage of Blue+ cells in the subset of mice circled in (A) was significantly different between the two groups of mice (Student's t test). (C) The distribution of cell types found within the organ (white bars, left y-axis) vs the distribution of cell types found in the Blue+ population (gray bars, right y-axis) for each infection condition was compared. (* P<0.05, ** P<0.01 and *** P<0.001). (TIF) [file ppat.1003415.s007.tif]
