# Supplementary material for: Adhesins and Host Serum Factors Drive Yop Translocation by Yersinia into Professional Phagocytes during Animal Infection
Source: PLoS Pathog. 2013 Jun 20;9(6):e1003415. doi: 10.1371/journal.ppat.1003415 (PMC3688556; doi:10.1371/journal.ppat.1003415)
Supplement: Table S1 — Strains and Plasmids. List of strains and plasmids constructed and/or used in this study. The Yptb strain from which the Yptb insert was cloned into pCVD442 is indicated in parenthesis. (DOCX) [file ppat.1003415.s008.docx]

**Supplementary Table 1. Strains and Plasmids**

| **Strains** | **Description** | **Source** |
| --- | --- | --- |
| *E. coli* | | |
| FM007 | DH5αλ pRK600 | [1] |
| FM036 | SM10λpir pSR47-YopETEM | [2] |
| FM038 | SM10λpir pCVD442-ΔyopB | [3] |
| FM040 | SM10λpir pCVD442-Δ*inv* | [4] |
| FM123 | SY327λpir pCVD442-Δ*ail* (IP2666) | This Study |
| FM135 | SY327λpir pCVD442-Δ*ail* (IP32953) | This Study |
| FM232 | SY327λpir pCVD442-*yadA* (IP2666) | This Study |
| FM339 | SY327λpir pCVD442-Δ*yadA* (IP2666) | This Study |
| FM361 | SY327λpir pCVD442-*ail* (YPIII, pIB1) | This Study |
| FM594 | XL1Blue pMMB207-inv | R. Isberg, Tufts University |
| *Y. pseudotuberculosis* | | |
| YED-32 | IP2666-NdeI pACYC184-GFP | [4] |
| FM033 | IP2666 ETEM | [2] |
| FM151 | IP2666 Δ*inv*Δy*adA* ETEM | This study |
| FM155 | IP2666 Δ*ail* ETEM | This study |
| FM163 | IP2666 Δ*ail*Δ*inv*Δ*yadA* ETEM | This study |
| FM175 | IP2666 Δ*inv* ETEM | This study |
| FM180 | IP2666 Δ*yadA* ETEM | This study |
| FM186 | IP2666 Δ*yopB* ETEM | This study |
| FM217 | IP2666 Δ*ail*Δ*inv* ETEM | This study |
| FM226 | IP2666 Δ*ail*Δ*yadA* ETEM | This study |
| FM283 | IP2666 Δ*ail*Δ*inv*Δ*yadA::yadA* ETEM | This study |
| FM290 | IP2666 Δ*ail*Δ*yadA::yadA* ETEM | This Study |
| FM325 | YPIII, pIB1 ETEM | This study |
| FM329 | YPIII, pIB1 Δ*yadA* ETEM | This study |
| FM335 | YPIII, pIB1 Δ*ail* ETEM | This study |
| FM369 | YPIII, pIB1 Y Δ*ail*Δ*yadA* ETEM | This study |
| FM402 | IP2666 Δ*ail*Δ*inv*Δ*yadA::ail* ETEM | This study |
| FM406 | IP2666 Δ*ail*Δ*yadA::ail* ETEM | This study |
| FM412 | IP32953 Δ*ail*Δ*inv*Δ*yadA* ETEM | This study |
| FM427 | YPIII, pIB1 Δ*inv*Δ*yadA* ETEM | This study |
| FM443 | IP2666 Δ*yopB* pACYC184-GFP | This study |
| FM445 | IP2666 Δ*ail*Δ*inv*Δ*yadA::yadA* pACYC184-GFP | This study |
| FM447 | IP2666 Δ*ail*Δ*yadA::yadA* pACYC184-GFP | This study |
| FM449 | IP2666 Δ*ail*Δ*inv*Δ*yadA::ail* pACYC184-GFP | This study |
| FM451 | IP2666 Δ*ail*Δ*yadA::ail* pACYC184-GFP | This study |
| FM475 | YPIII, pIB1 Δ*inv* ETEM | This study |
| FM477 | YPIII, pIB1 Δ*ail*Δ*inv* ETEM | This study |
| FM478 | YPIII, pIB1 Δ*ail*Δ*inv*Δ*yadA* ETEM | This study |
| FM483 | IP32953 Δ*yopB* ETEM | This study |
| FM511 | IP32953 Δ*ail*Δ*inv* ETEM | This study |
| FM513 | IP32953 Δ*inv* ETEM | This study |
| FM522 | YPIII Δ*yopB* ETEM | This study |
| FM612 | YPIII Δ*inv* ETEM pMMB207-inv | This study |
| FM616 | YPIII Δ*ail*Δ*inv*Δ*yadA* ETEM pMMB207-inv | This study |
| MKP032 | IP32953 ETEM | This study |
| MKP038 | IP32953 Δ*ail* ETEM | This study |
| MKP040 | IP32953 Δ*ail*Δ*yadA* ETEM | This study |
| MKP042 | IP32953 Δ*inv*Δ*yadA* ETEM | This study |
| MLF90 | IP32953 Δ*yadA* ETEM | This study |
| CG001 | IP2666 pACYC184-GFP | This study |
| CG003 | IP2666 Δ*ail* pACYC184-GFP | This study |
| CG005 | IP2666 Δ*yadA* pACYC184-GFP | This study |
| CG007 | IP2666 Δ*inv* pACYC184-GFP | This study |
| CG009 | IP2666 Δ*ail*Δ*yadA* pACYC184-GFP | This study |
| CG011 | IP2666 Δ*ail*Δ*inv* pACYC184-GFP | This study |
| CG013 | IP2666 Δ*inv*Δ*yadA* pACYC184-GFP | This study |
| CG015 | IP2666 Δ*ail*Δ*inv*Δ*yadA* pACYC184-GFP | This study |
