# Supplementary material for: Adhesins and Host Serum Factors Drive Yop Translocation by Yersinia into Professional Phagocytes during Animal Infection
Source: PLoS Pathog. 2013 Jun 20;9(6):e1003415. doi: 10.1371/journal.ppat.1003415 (PMC3688556; doi:10.1371/journal.ppat.1003415)
Supplement: Table S2 — List of Primers. List of primers used in this study to generate pCVD442 plasmids with Yptb inserts. (DOCX) [file ppat.1003415.s009.docx]

**Supplementary Table 2. List of Primers**

| **Primer name** | **Primer Sequence** |
| --- | --- |
| FM011 | 5’ – GATCCGTCGACcgtgtgaaacagaaagttctg |
| FM012 | 5’ – TTTGAATTCCGAAcataaaaaccatccagattgttataac |
| FM013 | 5’ – TTCGGAATTCAAAcggttctaacgtcctcctaac |
| FM014 | 5’ – GATCCGAGCTCgaaaaaaaataggtgaaatag |
| FM033 | 5’ – GATCCGCATGCcatccggtttgaggtgag |
| FM034 | 5’ – GATCCTCTAGAgtagcaaatatcggagagattg |
