# Supplementary material for: Adhesins and Host Serum Factors Drive Yop Translocation by Yersinia into Professional Phagocytes during Animal Infection
Source: PLoS Pathog. 2013 Jun 20;9(6):e1003415. doi: 10.1371/journal.ppat.1003415 (PMC3688556; doi:10.1371/journal.ppat.1003415)
Supplement: Methods S1 — Supporting Materials and Methods. (DOCX) [file ppat.1003415.s010.docx]

**Supplementary Methods**

**Strains and bacterial culture conditions**

Strains used for these studies are listed in Table S1. *Yptb* strains were cultured in 2XYT at 26°C overnight with aeration. For experiments with isolated splenocytes, strains were diluted 1:40 into 2XYT containing 5mM CaCl_2_, incubated at 26°C for 1.5h with aeration, followed by incubation at 37°C for 1.5h with aeration to induce the TTSS. To induce *invasin* expression in strains carrying the pMMB207-inv plasmid (gift from Ralph Isberg, Tufts University), IPTG was added to a final concentration of 0.2mM when cultures were switched to 37°C. Cultures for murine infections were grown as described previously [[1](#_ENREF_1)].

The *Δinv*, *ΔyadA* and *ΔinvΔyadA* mutants were described previously [[2](#_ENREF_2),[3](#_ENREF_3)]. The *ail* gene was deleted by allelic exchange [[4](#_ENREF_4)]. Overlapping PCR was used to generate fragments flanking the upstream and downstream regions of Ail. The primer pairs FM011/FM012, and FM013/FM014 (Table S2) were used to amplify the upstream and downstream regions from IP2666 or IP32953 genomic DNA, respectively. A second PCR was carried out to stitch together the overlapping PCR fragments using primers FM011 and FM014. The overlapping PCR product was cloned into pCVD442 using Sac I and Sal I restriction sites. The resulting plasmids, pCVD442-ailKO_IP2666_ and pCVD442-ailKO_IP32953_ (FM123 and FM135, respectively), were then transformed into *E. coli* SY327λpir. Allelic exchange through triparental mating of helper (*E. coli* DH5α/pRK600), donor (FM123 for IP2666 and YPIII or FM135 for IP32953) and recipient *Yptb* strains (IP2666, IP32953 or YPIII) was used to remove the coding region of *ail*. Deletion of *ail* was confirmed by PCR using primers FM011 and FM014 (Table S2).

To complement adhesin mutant strains with *invasin*, YPIII strains were transformed with the pMMB207-inv plasmid. To complement adhesin mutant strains with Ail or YadA, the *ail* and *yadA* genes were each amplified using primer pairs FM011/FM014 for *ail* and FM033/FM034 for *yadA*. The PCR products were cloned into pCVD442 using Sac I and Sal I restriction sites for *ail* or Sph I and Xba I sites for *yadA,* and the resulting plasmids, FM361 (*ail*) and FM232 (*yadA*), were then transformed into *E. coli* SY327λpir. Allelic exchange was used as described above to introduce *ail* and *yadA* genes into *Yptb* *Δail* and *ΔyadA* strains.

All ETEM expressing strains were generated as described previously by conjugating strain FM036 with each *Yptb* strain to cross the chimeric YopE-TEM gene into the *yopE* locus [[2](#_ENREF_2)]. All strains were tested by western blot analysis to demonstrate that ETEM, YopE and other Yops were expressed and secreted at normal levels [[2](#_ENREF_2),[5](#_ENREF_5)]. All GFP-expressing strains were constructed by transforming the pACYC184-GFP plasmid [[3](#_ENREF_3)] into the corresponding *Yptb* strains.

**Propidium Iodide Staining for Viability**

Splenocytes were isolated as described in the Materials and Methods. Splenocyte suspensions were incubated with Propidium iodide (PI) solution (1μg/ml) in FACS buffer for 30 minutes and then analyzed by flow cytometry on an LSRII FACS machine.

**Western Blot Analysis**

*Yptb* strains were grown as described in the figure legends and lysed in SDS sample buffer. 5x10^7^ cfu were separated on SDS-PAGE, transferred to polyvinyl difluoride (PVDF) membrane and probed with antibodies reactive against Ail or Invasin (gift from Ralph Isberg, Tufts University), or YadA (bA-17, Santa Cruz), followed by the horseradish peroxidase conjugated goat-anti-rabbit-IgG (Invitrogen), goat-anti-mouse-IgG (Invitrogen) or -rabbit-anti-goat-IgG (Invitrogen).

**References**

1. Logsdon LK, Mecsas J (2003) Requirement of the Yersinia pseudotuberculosis effectors YopH and YopE in colonization and persistence in intestinal and lymph tissues. Infect Immun 71: 4595-4607.

2. Harmon DE, Davis AJ, Castillo C, Mecsas J (2010) Identification and characterization of small-molecule inhibitors of Yop translocation in Yersinia pseudotuberculosis. Antimicrob Agents Chemother 54: 3241-3254.

3. Durand EA, Maldonado-Arocho FJ, Castillo C, Walsh RL, Mecsas J (2010) The presence of professional phagocytes dictates the number of host cells targeted for Yop translocation during infection. Cell Microbiol 12: 1064-1082.

4. Donnenberg MS, Kaper JB (1991) Construction of an eae deletion mutant of enteropathogenic Escherichia coli by using a positive-selection suicide vector. Infect Immun 59: 4310-4317.

5. Davis AJ, Mecsas J (2007) Mutations in the Yersinia pseudotuberculosis type III secretion system needle protein, YscF, that specifically abrogate effector translocation into host cells. J Bacteriol 189: 83-97.
